# Vitamin D Modulates Expression of the Airway Smooth Muscle Transcriptome in Fatal Asthma

**DOI:** 10.1371/journal.pone.0134057

**Published:** 2015-07-24

**Authors:** Blanca E. Himes, Cynthia Koziol-White, Martin Johnson, Christina Nikolos, William Jester, Barbara Klanderman, Augusto A. Litonjua, Kelan G. Tantisira, Kevin Truskowski, Kevin MacDonald, Reynold A. Panettieri, Scott T. Weiss

**Affiliations:** 1 Department of Biostatistics and Epidemiology, University of Pennsylvania, Philadelphia, PA, United States of America; 2 Airways Biology Initiative, Pulmonary, Allergy and Critical Care Division, University of Pennsylvania, Philadelphia, PA, United States of America; 3 Partners Personalized Medicine, Boston, MA, United States of America; 4 Channing Division of Network Medicine, Brigham and Women’s Hospital and Harvard Medical School, Boston, MA, United States of America; University of Giessen Lung Center, GERMANY

## Abstract

Globally, asthma is a chronic inflammatory respiratory disease affecting over 300 million people. Some asthma patients remain poorly controlled by conventional therapies and experience more life-threatening exacerbations. Vitamin D, as an adjunct therapy, may improve disease control in severe asthma patients since vitamin D enhances glucocorticoid responsiveness and mitigates airway smooth muscle (ASM) hyperplasia. We sought to characterize differences in transcriptome responsiveness to vitamin D between fatal asthma- and non-asthma-derived ASM by using RNA-Seq to measure ASM transcript expression in five donors with fatal asthma and ten non-asthma-derived donors at baseline and with vitamin D treatment. Based on a Benjamini-Hochberg corrected p-value <0.05, 838 genes were differentially expressed in fatal asthma vs. non-asthma-derived ASM at baseline, and vitamin D treatment compared to baseline conditions induced differential expression of 711 and 867 genes in fatal asthma- and non-asthma-derived ASM, respectively. Functional gene categories that were highly represented in all groups included extracellular matrix, and responses to steroid hormone stimuli and wounding. Genes differentially expressed by vitamin D also included cytokine and chemokine activity categories. Follow-up qPCR and individual analyte ELISA experiments were conducted for four cytokines (i.e. CCL2, CCL13, CXCL12, IL8) to measure TNFα-induced changes by asthma status and vitamin D treatment. Vitamin D inhibited TNFα-induced IL8 protein secretion levels to a comparable degree in fatal asthma- and non-asthma-derived ASM even though IL8 had significantly higher baseline levels in fatal asthma-derived ASM. Our findings identify vitamin D-specific gene targets and provide transcriptomic data to explore differences in the ASM of fatal asthma- and non-asthma-derived donors.

## Introduction

Asthma, a chronic inflammatory respiratory disease, is defined by increased airway responsiveness and inflammation to specific environmental stimuli [1]. Clinical therapy following established guidelines successfully controls asthma symptoms in most patients [2]. Such therapy includes use of glucocorticoids, anti-inflammatory medications [3], and β_2_-agonists that promote bronchodilation [4]. Up to 5% of asthma patients, however, are estimated to inadequately respond to conventional approaches, and consequently, are at greater risk of suffering life-threatening asthma exacerbations [5]. While patients with severe, refractory disease represent a heterogeneous group, features shared by most are glucocorticoid insensitivity and, in part, irreversible airflow obstruction [6].

Low vitamin D levels are associated with decreased lung function and increased asthma exacerbations and medication use, although it is unclear whether there is a causal relationship among these observations or whether asthma patients can benefit from vitamin D supplementation [7]. Mechanisms by which vitamin D may ameliorate the asthma diathesis include modulating inflammation by increasing CD4+ T cell synthesis of IL-10 [8], suppressing Th17 cytokines in PBMCs [9], and increasing the number of regulatory T cells [10]. These anti-inflammatory effects of vitamin D extend to structural cells, including the airway smooth muscle (ASM). For example, vitamin D reduced TNFα-induced CCL5 (RANTES) and CXCL10 (IP-10) secretion in ASM cells, while secretion of CX3CL1 (fractalkine), which is glucocorticoid-insensitive, was also decreased by vitamin D treatment [11]. Others found that vitamin D decreased expression of *KPNA4* (importin α3), a signaling molecule for NFκB-induced inflammation in the ASM [12]. Vitamin D anti-inflammatory effects are, in part, mediated by enhancing the action of glucocorticoids, suggesting that vitamin D supplementation may benefit patients with glucocorticoid-insensitive asthma [7].

Airway remodeling, an important feature of severe persistent asthma that includes ASM hyperplasia, is modulated by vitamin D since growth factor-induced ASM proliferation was inhibited by vitamin D treatment [13,14]. Children with severe, therapy-resistant asthma also manifested lower levels of vitamin D than children with moderate asthma; endobronchial biopsies showed an inverse relationship of vitamin D levels and ASM mass, but not epithelial shedding, reticular basement membrane thickness, or airway inflammation measures [15]. Accordingly, the beneficial vitamin D effects on the ASM may extend beyond the enhancement of glucocorticoid actions since glucocorticoids have little effect on airway remodeling or ASM proliferation [11].

Vitamin D (1,25(OH)_2_D_3_) plays a pivotal role in the regulation of gene transcription [16]. Consequently, the study of vitamin D effects on gene transcription in multiple cell types may reveal changes in signaling pathways that are relevant to specific diseases. For example, a microarray study that used vitamin D-treated ASM cells from a single male donor without asthma identified transcription changes in gene categories that might influence airway remodeling, including morphogenesis, cell growth and cell survival [17]. Recent advances in sequencing technologies provide comprehensive and in-depth characterization of transcriptomes via RNA-Seq [18]. Compared to the use of microarrays, RNA-Seq offers advantages by examining greater numbers of RNA species over a wider dynamic range of signal [19].

In this study, we used RNA-Seq to identify differences between the transcriptomes of ASM derived from fatal asthma and non-asthma donors to vitamin D treatment, and we also specifically examined four cytokines (i.e. *CCL2*, *CCL13*, *CXCL12*, *IL8*) whose expression differed between fatal asthma- and non-asthma-derived ASM at baseline and with vitamin D treatment.

## Results

### Transcriptomic Differences Between Fatal Asthma- and Non-Asthma-Derived ASM

After quality control processing, 30 samples were selected for differential expression analysis. Such samples corresponded to 5 fatal asthma and 10 non-asthma donors with vitamin D or control vehicle treatment [Table 1]. Comparison of fatal asthma-derived vs. non-asthma-derived ASM at baseline revealed that 838 genes were significantly differentially expressed according to a 0.05 false discovery rate threshold (i.e. q-value < 0.05) [Fig A in S1 Fig]. Differentially expressed genes were prioritized by (1) selecting the 370 genes with the lowest q-value of 1.92E-03, (2) selecting the subset of 192 of these genes with magnitude of Log_2_ fold-change >1.3, and (3) dropping genes for which at least two individual samples did not have FPKM values >5 [Table 2]. Among the top genes are ones that have been previously related to asthma (e.g., *APOE* [20], *CHI3L1* [21], *EGR1* [22], *MYH11* [23], *OXTR* [24], *SERPINB2* [25,26], *SOCS3* [27]) and corticosteroid-resistant asthma (e.g., *FOS* [28]) though not necessarily in humans or via changes of the ASM. Additionally, there were many novel genes that have not been previously related to asthma phenotypes. To gain a better sense of the biological functions represented by the 838 differentially expressed genes, we performed an ontological category enrichment analysis using the NIH DAVID tool [29]. There were 24 annotation clusters with enrichment scores >2.50 [S1 Table]. Among these clusters, some of the individual enrichment categories had Benjamini-Hochberg adjusted p-values < 0.05 and referred to specific biological structures or processes that are associated with asthma: immunoglobulin domain, lung development, response to steroid hormone stimulus, response to wounding, extracellular matrix, and response to oxidative stress. S2 Table contains the gene members for these categories and two that were significant in vitamin D treated ASM cells vs. ASM cells at baseline.

**Table 1:** Characteristics of ASM donors. All donors were white non-smokers. There were no significant differences between sex, age, or BMI of fatal asthma- vs. non-asthma-derived donors. Medical examiner ruled cause of death for fatal asthma donors was “Asthma Attack/Anoxia,” or a significant asthma event is listed as preceding death.

	Fatal Asthma (N = 5)	Non-Asthma (N = 10)
Sex		
Male	2	6
Female	3	4
Age		
Mean (SD)	31.2 (20.1)	33.1 (15.2)
[Range]	[9, 59]	[17, 52]
BMI		
Mean (SD)	27.4 (6.5)	27.4 (6.5)
[Range]	[17.9, 34.5]	[21.5, 40.5]
Cause of Death		
CVA	-	2
Head Trauma	-	4
Anoxia	-	3
AVM rupture	-	1

**Table 2:** Top differentially expressed genes in fatal asthma- vs. non-asthma-derived ASM at baseline. FPKM = fragments per kilobase of transcript per million mapped reads. All genes in list have q-value = 1.92E-03, magnitude Log_2_(Fold Change) >1.30, and at least two individual samples with FPKM >5.

Gene	Locus	Mean FPKM Non-Asthma	Mean FPKM Fatal Asthma	Log2[Fold Change]
*SERPINB2*	chr18:61554938–61571124	0.41	39.18	6.57
*FOS*	chr14:75745480–75748937	56.13	0.66	-6.42
*HBD*	chr11:5254058–5255858	0.94	24.31	4.70
*EGR1*	chr5:137801180–137805004	232.04	11.36	-4.35
*MYH11*	chr16:15737123–15950887	8.15	0.46	-4.14
*CHI3L1*	chr1:203148058–203155922	57.11	4.21	-3.76
*SFRP2*	chr4:154701741–154710228	7.44	0.63	-3.57
*DES*	chr2:220283098–220291461	13.82	1.29	-3.42
*HLA-C*	chr6:31236525–31239913	18.62	1.85	-3.33
*EGR2*	chr10:64571755–64578927	2.99	0.36	-3.07
*IFI6*	chr1:27992571–27998724	57.11	461.09	3.01
*IFI27*	chr14:94577078–94583033	8.88	65.13	2.88
*A2M*	chr12:9217772–9268558	341.16	46.75	-2.87
*EGR3*	chr8:22545173–22550815	3.72	0.54	-2.79
*SCUBE3*	chr6:35182189–35218609	1.02	6.94	2.76
*TINAGL1*	chr1:32042085–32053287	1.69	10.99	2.70
*PDLIM3*	chr4:186421814–186456712	4.63	0.73	-2.67
*SYPL2*	chr1:110009099–110024764	1.14	7.03	2.63
*RARRES1*	chr3:158414896–158450275	119.42	20.05	-2.57
*PSG4*	chr19:43696853–43709790	23.85	138.94	2.54
*SOCS3*	chr17:76352858–76356158	111.55	20.00	-2.48
*APOE*	chr19:45409038–45412650	99.29	18.03	-2.46
*B4GALNT4*	chr11:369794–382117	7.42	1.41	-2.39
*TM4SF20*	chr2:228226873–228244022	0.75	3.93	2.38
*NPAS1*	chr19:47524142–47549017	0.69	3.51	2.35
*JUNB*	chr19:12902309–12904125	132.22	26.05	-2.34
*PTGS2*	chr1:186640943–186649559	5.40	1.06	-2.34
*MX1*	chr21:42792519–42831141	3.79	18.85	2.32
*IL8*	chr4:74606222–74609433	0.87	4.31	2.31
*PPP1R1B*	chr17:37783176–37792878	7.63	1.54	-2.31
*IGFBP7*	chr4:57845108–58071465	141.30	673.27	2.25
*ACTG2*	chr2:74120092–74146780	41.93	8.95	-2.23
*HLA-B*	chr6:31321648–31324989	42.92	9.34	-2.20
*TEK*	chr9:27109146–27230172	1.28	5.82	2.19
*OXTR*	chr3:8792094–8811300	5.32	23.54	2.15
*SCN4B*	chr11:118004091–118023630	4.44	1.01	-2.14
*CCL13*	chr17:32683470–32685629	1.31	5.62	2.10
*NDNF*	chr4:121956781–121993673	4.00	0.96	-2.06
*TFAP2A*	chr6:10396915–10419797	1.73	7.13	2.05
*NR4A1*	chr12:52416615–52453291	17.27	4.21	-2.04
*OLR1*	chr12:10310898–10324790	2.49	10.19	2.03
*IER2*	chr19:13261281–13265718	68.06	16.68	-2.03
*CPNE7*	chr16:89642175–89663654	1.61	6.27	1.96
*CERS4*	chr19:8274216–8327304	10.96	2.86	-1.94
*CECR1*	chr22:17660191–17690779	13.18	3.44	-1.94
*CES1*	chr16:55836763–55867075	59.97	15.70	-1.93
*ISG15*	chr1:948846–949919	28.51	108.61	1.93
*SPON1*	chr11:13984183–14289679	8.25	2.17	-1.93
*PSG5*	chr19:43671894–43690688	50.63	191.94	1.92
*FNDC1*	chr6:159590428–159693140	4.85	1.29	-1.91
*FMO2*	chr1:171154387–171181822	14.66	3.93	-1.90
*SERPINA3*	chr14:95078713–95090390	29.20	7.89	-1.89
*PEAR1*	chr1:156863522–156886226	2.69	9.73	1.86
*RASL12*	chr15:65337707–65360388	24.77	6.86	-1.85
*ADAMTS5*	chr21:28290230–28339439	5.87	20.98	1.84
*ABCG1*	chr21:43619798–43717354	6.48	1.83	-1.82
*GDF10*	chr10:48425787–48439138	102.61	29.10	-1.82
*SGCG*	chr13:23755059–23899304	1.82	6.42	1.82
*AEBP1*	chr7:44143959–44154159	144.69	41.17	-1.81
*CCKAR*	chr4:26483017–26492042	31.20	9.09	-1.78
*SHC2*	chr19:416582–460996	10.66	3.11	-1.78
*HSD17B6*	chr12:57157107–57181574	7.60	25.93	1.77
*ALPK2*	chr18:56148481–56296189	2.15	7.31	1.76
*PSG1*	chr19:43370612–43383871	4.13	13.95	1.76
*GPRC5A*	chr12:13043955–13066600	6.30	21.17	1.75
*LOC645638*	chr17:58160926–58165828	11.94	39.70	1.73
*PTGDS*	chr9:139871955–139876194	433.66	134.15	-1.69
*MYOC*	chr1:171604556–171621773	13.18	4.13	-1.67
*MFAP5*	chr12:8798539–8815433	26.25	82.96	1.66
*TBX4*	chr17:59533806–59561664	18.10	5.73	-1.66
*CHI3L2*	chr1:111770280–111786062	46.33	14.69	-1.66
*BAMBI*	chr10:28966423–28971868	5.68	17.73	1.64
*GNG2*	chr14:52327021–52436518	5.04	1.63	-1.63
*MARCH4*	chr2:217122584–217236750	3.17	9.32	1.56
*DMKN*	chr19:35988118–36004560	39.40	13.56	-1.54
*CPA4*	chr7:129932973–129964020	3.08	8.90	1.53
*KRT7*	chr12:52626953–52642709	13.56	39.15	1.53
*COL14A1*	chr8:121137351–121384273	35.38	12.27	-1.53
*PLCB4*	chr20:9049700–9461462	3.51	10.06	1.52
*SLC2A1*	chr1:43391045–43449029	33.40	95.72	1.52
*STEAP3*	chr2:119981383–120023227	4.57	12.99	1.51
*GLT25D2*	chr1:183904965–184006863	2.27	6.43	1.50
*TFPI2*	chr7:93515744–93520065	4.81	13.47	1.49
*PCSK2*	chr20:17206751–17465222	7.50	2.68	-1.49
*DKK1*	chr10:54074040–54077417	17.41	47.95	1.46
*ITGA6*	chr2:173292313–173371181	1.81	4.98	1.46
*CLEC3B*	chr3:45067758–45077563	47.23	129.11	1.45
*FGFR4*	chr5:176513920–176525126	6.56	2.43	-1.43
*CSRNP1*	chr3:39183341–39195102	5.77	2.15	-1.42
*SLC7A5*	chr16:87863628–87903100	14.64	39.01	1.41
*GPC3*	chrX:132669775–133119673	29.19	11.03	-1.40
*WISP2*	chr20:43343884–43356452	46.81	17.71	-1.40
*RGMA*	chr15:93586635–93632443	11.83	4.53	-1.38
*TUFT1*	chr1:151512780–151556059	4.50	11.74	1.38
*LOC728392*	chr17:5402746–5404319	29.62	76.86	1.38
*F3*	chr1:94994731–95007413	18.63	48.32	1.37
*SLC7A11*	chr4:138948576–139163503	5.71	14.80	1.37
*SERPINE1*	chr7:100770378–100782547	118.50	306.35	1.37
*PDPN*	chr1:13910251–13944452	9.84	3.82	-1.37
*SULF2*	chr20:46286149–46415360	33.96	13.21	-1.36
*IL32*	chr16:3115312–3119668	25.90	66.18	1.35
*DCHS1*	chr11:6642557–6677074	6.65	2.62	-1.34
*CHRM2*	chr7:136553398–136849088	15.63	39.61	1.34
*TLE2*	chr19:2997635–3047633	6.74	2.67	-1.34
*BST2*	chr19:17513754–17516384	8.51	21.35	1.33
*COLEC12*	chr18:319354–500729	68.62	27.44	-1.32
*RSPO1*	chr1:38076950–38100595	7.77	3.11	-1.32
*ARHGAP28*	chr18:6834431–6915712	5.49	2.21	-1.32
*PTPRD*	chr9:8314245–10612723	4.92	1.99	-1.31
*SNHG5*	chr6:86386724–86388451	488.49	198.98	-1.30

### Influence of Vitamin D on the Transcriptome of Fatal Asthma- and Non-Asthma-Derived ASM

Comparison of genes expressed in ASM when treated with vitamin D vs. baseline revealed that 867 genes were differentially expressed in non-asthma-derived ASM, while 711 genes were differentially expressed in fatal asthma-derived ASM, in response to vitamin D treatment [Figs B and C in S1 Fig]. A subset of 307 genes was vitamin D responsive in both fatal asthma- and non-asthma-derived ASM. As for the fatal asthma- vs. non-asthma-derived comparison, differentially expressed genes were prioritized by (1) selecting the genes with the lowest q-value of 1.92E-03 (n = 394 for non-asthma-derived ASM; n = 345 for fatal asthma-derived ASM), (2) selecting the subset of these genes with magnitude of Log_2_ fold-change >1.3 (n = 105 for non-asthma-derived ASM; n = 214 for fatal asthma-derived ASM), and (3) dropping genes for which at least two individual samples did not have FPKM values >5 [Tables 3 and 4].

**Table 3:** Top differentially expressed genes in non-asthma-derived ASM at baseline vs. when treated with vitamin D. FPKM = fragments per kilobase of transcript per million mapped reads. All genes in list have q-value = 1.92E-03, magnitude Log_2_(Fold Change) >1.30, and at least two individual samples with FPKM >5.

Gene	Locus	Mean FPKM Non-Asthma Baseline	Mean FPKM Non-Asthma Vit D	Log2[Fold Change]
*CYP24A1*	chr20:52769987–52790516	0.25	157.66	9.30
*CXCL6*	chr4:74702272–74704477	9.35	0.35	-4.73
*CRIP1*	chr14:105953256–105955124	3.06	41.77	3.77
*FOS*	chr14:75745480–75748937	56.13	4.84	-3.53
*CXCL1*	chr4:74735108–74737019	7.64	0.66	-3.53
*CYP3A5*	chr7:99245812–99277621	0.22	2.41	3.42
*HBA2*	chr16:222845–223709	7.87	0.74	-3.42
*G0S2*	chr1:209848669–209849735	6.09	61.25	3.33
*IGFLR1*	chr19:36230150–36233351	1.85	16.73	3.18
*CILP*	chr15:65488336–65503840	3.22	24.30	2.91
*PTGS2*	chr1:186640943–186649559	5.40	0.79	-2.76
*THBD*	chr20:23026269–23030301	0.31	1.97	2.69
*HBB*	chr11:5246695–5248301	12.56	1.98	-2.66
*EGR1*	chr5:137801180–137805004	232.04	37.68	-2.62
*TM7SF2*	chr11:64879340–64883707	4.60	27.11	2.56
*IDH2*	chr15:90627211–90645708	21.35	120.25	2.49
*CCL7*	chr17:32597239–32599256	3.34	0.65	-2.35
*CCKAR*	chr4:26483017–26492042	31.20	6.64	-2.23
*NGEF*	chr2:233743395–233877951	0.32	1.49	2.20
*C15orf48*	chr15:45722762–45725647	1.16	5.28	2.19
*LGALS9*	chr17:25958173–25976586	1.40	6.01	2.10
*SLPI*	chr20:43880878–43883206	3.13	12.17	1.96
*EGR2*	chr10:64571755–64578927	2.99	0.81	-1.89
*TGFB3*	chr14:76424441–76448092	3.46	12.80	1.89
*EFTUD1*	chr15:82422560–82555104	5.55	20.44	1.88
*OAF*	chr11:120081746–120100650	80.02	290.60	1.86
*CCL11*	chr17:32612686–32615199	45.36	12.76	-1.83
*FZD8*	chr10:35927176–35930362	1.21	4.21	1.80
*SLC1A3*	chr5:36606456–36688436	1.66	5.72	1.78
*NFKBIZ*	chr3:101498028–101579869	11.86	3.54	-1.75
*IL13RA2*	chrX:114238537–114252207	7.77	2.33	-1.73
*EGR3*	chr8:22545173–22550815	3.72	1.12	-1.73
*HMCN1*	chr1:185703682–186446655	2.43	7.97	1.71
*NR4A1*	chr12:52416615–52453291	17.27	5.30	-1.70
*CCL2*	chr17:32582295–32584220	166.20	51.14	-1.70
*SFRP2*	chr4:154701741–154710228	7.44	24.17	1.70
*SYT7*	chr11:61281187–61348344	2.66	8.53	1.68
*SPINT2*	chr19:38755097–38783254	1.53	4.83	1.65
*DUSP5*	chr10:112257624–112271302	14.73	4.76	-1.63
*FER1L6*	chr8:124864226–125132302	0.99	3.05	1.62
*ACAN*	chr15:89346673–89418585	0.70	2.15	1.62
*FAIM2*	chr12:50260678–50297760	2.62	7.92	1.60
*CYR61*	chr1:86046443–86049648	370.72	128.01	-1.53
*SOCS3*	chr17:76352858–76356158	111.55	38.80	-1.52
*SLC14A1*	chr18:43304091–43332485	1.48	4.20	1.50
*H19*	chr11:2016405–2019065	13.48	37.13	1.46
*SHISA3*	chr4:42399855–42404504	1.58	4.34	1.46
*OSR1*	chr2:19551245–19558372	20.37	55.38	1.44
*CYP3A7*	chr7:99282301–99332819	1.34	3.63	1.44
*SLMO1*	chr18:12407894–12432236	3.08	8.34	1.44
*SORCS2*	chr4:7194373–7744564	0.94	2.51	1.42
*CD248*	chr11:66081957–66084515	286.58	764.29	1.42
*NTRK2*	chr9:87283465–87638505	1.08	2.88	1.41
*SEMA3B*	chr3:50305039–50314572	29.16	76.45	1.39
*CNIH3*	chr1:224804178–224928249	2.87	7.44	1.37
*ALPL*	chr1:21835857–21904905	8.46	21.91	1.37
*DDIT4*	chr10:74033676–74035797	37.93	98.15	1.37
*PRSS35*	chr6:84222193–84235421	7.74	3.00	-1.37
*PSG1*	chr19:43370612–43383871	4.13	1.61	-1.36
*ITPR1*	chr3:4535031–4889524	5.69	14.44	1.34
*SMTNL2*	chr17:4487275–4511614	1.69	4.29	1.34
*APBB1IP*	chr10:26727265–26856732	5.15	12.84	1.32
*MAPK13*	chr6:36098261–36107842	2.90	7.21	1.31
*RHOU*	chr1:228780393–228882416	3.14	7.78	1.31
*MYOC*	chr1:171604556–171621773	13.18	5.37	-1.30
*SCARA5*	chr8:27727398–27850369	3.20	7.86	1.30

**Table 4:** Top differentially expressed genes in fatal asthma-derived ASM at baseline vs. when treated with vitamin D. FPKM = fragments per kilobase of transcript per million mapped reads. All genes in list have q-value = 1.92E-03, magnitude Log_2_(Fold Change) >1.30, and at least two individual samples with FPKM >5.

Gene	Locus	Mean FPKM Fatal Asthma Baseline	Mean FPKM Fatal Asthma Vit D	Log2[Fold Change]
*HBD*	chr11:5254058–5255858	24.31	0.58	-5.39
*SERPINB2*	chr18:61554938–61571124	39.18	1.10	-5.16
*IL8*	chr4:74606222–74609433	4.31	0.16	-4.79
*CXCL1*	chr4:74735108–74737019	14.18	0.68	-4.37
*CILP*	chr15:65488336–65503840	1.31	24.45	4.22
*RMRP*	chr9:35657747–35658015	4.64	81.69	4.14
*RPPH1*	chr14:20811229–20811570	2.21	30.69	3.80
*CYP3A7*	chr7:99282301–99332819	0.62	8.02	3.69
*IGFLR1*	chr19:36230150–36233351	1.22	14.72	3.59
*CHRDL2*	chr11:74407473–74442186	0.76	7.35	3.28
*CRIP1*	chr14:105953256–105955124	3.87	36.71	3.25
*APLN*	chrX:128779235–128788933	0.91	7.46	3.04
*IFI6*	chr1:27992571–27998724	461.09	56.11	-3.04
*MX1*	chr21:42792519–42831141	18.85	2.31	-3.03
*SLC1A3*	chr5:36606456–36688436	0.91	7.38	3.02
*G0S2*	chr1:209848669–209849735	4.72	37.54	2.99
*IGF1*	chr12:102789644–102874378	2.82	20.63	2.87
*TM4SF20*	chr2:228226873–228244022	3.93	0.63	-2.65
*FOS*	chr14:75745480–75748937	0.66	4.00	2.61
*SYT7*	chr11:61281187–61348344	1.24	7.24	2.54
*PCSK2*	chr20:17206751–17465222	2.68	15.36	2.52
*CD14*	chr5:140011312–140013286	0.89	5.04	2.51
*IFI27*	chr14:94577078–94583033	65.13	11.57	-2.49
*SYPL2*	chr1:110009099–110024764	7.03	1.33	-2.40
*ACAN*	chr15:89346673–89418585	12.80	2.43	-2.40
*AOC3*	chr17:41003200–41010140	2.45	12.69	2.38
*IDH2*	chr15:90627211–90645708	24.40	118.59	2.28
*TM7SF2*	chr11:64879340–64883707	4.97	23.07	2.21
*CCL2*	chr17:32582295–32584220	300.27	65.12	-2.21
*APOE*	chr19:45409038–45412650	18.03	82.34	2.19
*ELN*	chr7:73442426–73484236	85.14	388.52	2.19
*MAP2*	chr2:210288770–210598834	11.95	2.68	-2.16
*LEPREL1*	chr3:189674516–189840226	4.64	1.05	-2.15
*OAF*	chr11:120081746–120100650	78.06	342.82	2.13
*CCL11*	chr17:32612686–32615199	67.55	15.46	-2.13
*COL14A1*	chr8:121137351–121384273	12.27	53.43	2.12
*VEGFA*	chr6:43737945–43754223	32.93	139.20	2.08
*EFTUD1*	chr15:82422560–82555104	5.67	23.85	2.07
*GPRC5A*	chr12:13043955–13066600	21.17	5.04	-2.07
*ANXA3*	chr4:79472741–79531605	5.30	1.26	-2.07
*IFIT1*	chr10:91152321–91163744	30.48	7.31	-2.06
*RARRES2*	chr7:150035406–150038763	156.60	651.36	2.06
*PSG5*	chr19:43671894–43690688	191.94	48.03	-2.00
*CES1*	chr16:55836763–55867075	15.70	62.37	1.99
*COL5A3*	chr19:10070236–10121147	3.01	11.75	1.97
*TPD52L1*	chr6:125474878–125584644	43.06	11.17	-1.95
*ITPR1*	chr3:4535031–4889524	3.83	14.71	1.94
*APOD*	chr3:195295572–195311076	13.81	52.99	1.94
*ISG15*	chr1:948846–949919	108.61	28.92	-1.91
*ALPL*	chr1:21835857–21904905	12.61	47.26	1.91
*PRSS35*	chr6:84222193–84235421	16.34	4.36	-1.91
*SPON1*	chr11:13984183–14289679	2.17	8.12	1.90
*ACTG2*	chr2:74120092–74146780	8.95	33.49	1.90
*EPSTI1*	chr13:43462121–43566377	7.54	2.02	-1.90
*RHOU*	chr1:228780393–228882416	2.03	7.50	1.89
*OLR1*	chr12:10310898–10324790	10.19	2.78	-1.87
*ABCA9*	chr17:66970772–67057136	5.06	18.18	1.85
*CD274*	chr9:5450502–5470567	1.21	4.33	1.84
*RASL12*	chr15:65337707–65360388	6.86	24.44	1.83
*FAIM2*	chr12:50260678–50297760	1.39	4.96	1.83
*NOV*	chr8:120428551–120436678	23.67	6.68	-1.83
*TGFB3*	chr14:76424441–76448092	3.09	10.85	1.81
*PSG1*	chr19:43370612–43383871	13.95	3.98	-1.81
*HTRA3*	chr4:8271488–8308838	10.31	35.91	1.80
*WISP2*	chr20:43343884–43356452	17.71	61.21	1.79
*GDF6*	chr8:97154557–97173020	0.89	3.07	1.78
*SCUBE3*	chr6:35182189–35218609	6.94	2.02	-1.78
*CXCL12*	chr10:44865604–44880545	294.15	1009.51	1.78
*AEBP1*	chr7:44143959–44154159	41.17	139.15	1.76
*IFI44*	chr1:79115476–79129763	11.90	3.54	-1.75
*DMKN*	chr19:35988118–36004560	13.56	45.33	1.74
*PTGES*	chr9:132500614–132515344	25.50	7.71	-1.73
*SBSN*	chr19:36014268–36019253	4.46	14.73	1.72
*A2M*	chr12:9217772–9268558	46.75	154.00	1.72
*BAMBI*	chr10:28966423–28971868	17.73	5.39	-1.72
*TFPI2*	chr7:93515744–93520065	13.47	4.09	-1.72
*PSG4*	chr19:43696853–43709790	138.94	42.51	-1.71
*GDF10*	chr10:48425787–48439138	29.10	93.77	1.69
*RGMA*	chr15:93586635–93632443	4.53	14.60	1.69
*RSPO1*	chr1:38076950–38100595	3.11	9.96	1.68
*FNDC1*	chr6:159590428–159693140	1.29	4.13	1.68
*DDIT4*	chr10:74033676–74035797	44.62	141.01	1.66
*EGR1*	chr5:137801180–137805004	11.36	35.60	1.65
*LOC645638*	chr17:58160926–58165828	39.70	12.68	-1.65
*TGFB2*	chr1:218517537–218617961	3.68	11.52	1.64
*BHLHE40*	chr3:5021096–5026865	7.87	24.35	1.63
*TRIB3*	chr20:361307–378203	15.39	47.17	1.62
*IL13RA2*	chrX:114238537–114252207	10.77	3.54	-1.61
*ZFP36*	chr19:39897486–39900045	50.83	154.60	1.60
*BDKRB1*	chr14:96722546–96731100	28.88	9.64	-1.58
*SULF2*	chr20:46286149–46415360	13.21	39.57	1.58
*PDPN*	chr1:13910251–13944452	3.82	11.40	1.58
*HGF*	chr7:81331443–81399452	30.12	10.18	-1.57
*SERPINA3*	chr14:95078713–95090390	7.89	23.20	1.56
*SIX1*	chr14:61111416–61116155	6.62	19.44	1.56
*CHI3L2*	chr1:111770280–111786062	14.69	43.14	1.55
*GPC3*	chrX:132669775–133119673	11.03	32.23	1.55
*SIK1*	chr21:44834397–44847002	2.52	7.29	1.53
*IFIT3*	chr10:91087601–91100725	37.15	12.95	-1.52
*TOX*	chr8:59717976–60031767	5.66	1.98	-1.52
*ALDH1A1*	chr9:75515577–75568233	24.38	8.56	-1.51
*PLAU*	chr10:75669726–75682535	34.94	12.30	-1.51
*PDGFRL*	chr8:17433941–17500642	9.04	25.63	1.50
*RCAN1*	chr21:35888783–35987382	56.39	19.95	-1.50
*KIT*	chr4:55524094–55606881	7.00	2.48	-1.50
*TNFAIP3*	chr6:138188580–138204449	6.55	2.33	-1.49
*PTGDS*	chr9:139871955–139876194	134.15	376.14	1.49
*NQO1*	chr16:69743303–69760533	146.93	52.45	-1.49
*AK5*	chr1:77747661–78025654	8.17	2.92	-1.48
*ARHGAP28*	chr18:6834431–6915712	2.21	6.16	1.48
*EML3*	chr11:62369690–62382592	15.39	42.52	1.47
*PRPS1*	chrX:106871653–106894256	48.46	17.67	-1.46
*GCNT4*	chr5:74323288–74326724	3.88	1.42	-1.45
*OSR2*	chr8:99956630–99964326	96.60	262.33	1.44
*MAP2K3*	chr17:21187967–21218551	50.93	18.95	-1.43
*MREG*	chr2:216807313–216878346	1.92	5.15	1.42
*SPRY1*	chr4:124317955–124324909	7.20	19.31	1.42
*MIR210HG*	chr11:565656–568457	2.99	8.02	1.42
*EGFL6*	chrX:13587693–13651694	10.10	27.04	1.42
*SDK1*	chr7:3341079–4308631	3.00	8.00	1.41
*EDNRA*	chr4:148402068–148466106	1.71	4.55	1.41
*RARRES1*	chr3:158414896–158450275	20.05	53.09	1.40
*LIF*	chr22:30636441–30642796	4.79	1.81	-1.40
*ID3*	chr1:23884420–23886285	16.08	6.11	-1.40
*IL32*	chr16:3115312–3119668	66.18	25.59	-1.37
*PCOLCE2*	chr3:142536701–142608045	44.82	17.33	-1.37
*BST2*	chr19:17513754–17516384	21.35	8.27	-1.37
*TRNP1*	chr1:27320194–27327377	44.79	17.38	-1.37
*NCEH1*	chr3:172348434–172429008	6.67	2.59	-1.36
*ASS1*	chr9:133320093–133376661	75.65	29.42	-1.36
*C7*	chr5:40909598–40983042	6.62	2.58	-1.36
*DKK1*	chr10:54074040–54077417	47.95	18.78	-1.35
*SSX2IP*	chr1:85109389–85156240	7.20	2.82	-1.35
*GCLM*	chr1:94352589–94375012	44.79	17.60	-1.35
*SEMA3A*	chr7:83587658–83824217	10.51	4.17	-1.33
*ATP8B1*	chr18:55297533–55470327	24.99	9.96	-1.33
*PKDCC*	chr2:42275160–42285668	31.61	12.62	-1.32
*DUSP5*	chr10:112257624–112271302	11.06	4.41	-1.32
*GREM2*	chr1:240652872–240775462	8.10	3.24	-1.32
*NDRG1*	chr8:134249413–134309547	58.01	144.57	1.32
*SH2D4A*	chr8:19171080–19253729	25.97	10.50	-1.31
*ADAMTS15*	chr11:130318868–130346539	6.27	15.51	1.31
*NBL1*	chr1:19923470–19984949	165.17	407.92	1.30

To gain a better sense of biological functions represented by the differentially expressed genes, we performed an ontological category enrichment analysis using the NIH DAVID tool [29]. For the non-asthma-derived ASM at baseline vs. vitamin D treated comparison, there with 24 annotation clusters with enrichment scores >2.50 [S3 Table]. For the fatal asthma-derived ASM at baseline vs. vitamin D treated comparison, there with 21 annotation clusters with enrichment scores >2.50 [S4 Table]. Some of the individual asthma-related enrichment categories within clusters with Benjamini-Hochberg adjusted p-values < 0.05 were also present in the fatal asthma- vs. non-asthma-derived comparison: extracellular matrix, immunoglobulin domain, response to steroid hormone stimulus, and response to wounding [S5 and S6 Tables], although some genes within categories differed. The oxidative stress and lung development categories were still significant for the non-asthma-derived ASM treated with vitamin D vs. baseline but not for the fatal asthma-derived ASM treated with vitamin D vs. baseline. Two asthma-related categories that were not present in the fatal asthma- vs. non-asthma-derived ASM comparison were significant in both the vitamin D treated vs. baseline comparisons: cytokine activity and chemokine activity [S5 and S6 Tables].

### TNFα Responsiveness of Select Cytokines Measured by q-PCR

**Fig 1:**
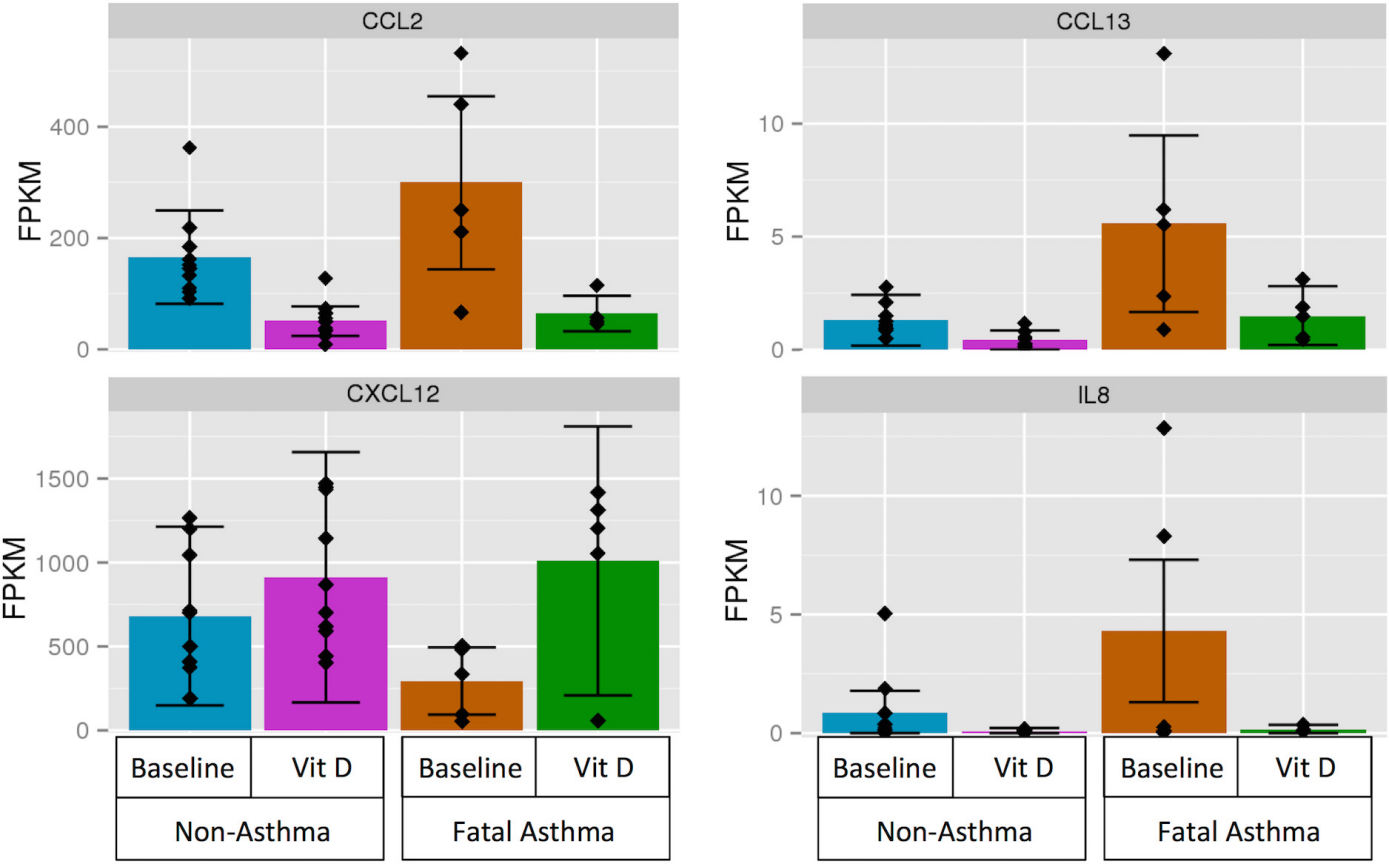
RNA-Seq results expressed as FPKM for four cytokine genes (i.e., *CCL2*, *CCL13*, *CXCL12*, *IL8*) by condition status. Table 5 lists q-values and fold changes among comparison groups for these genes.

**Fig 2:**
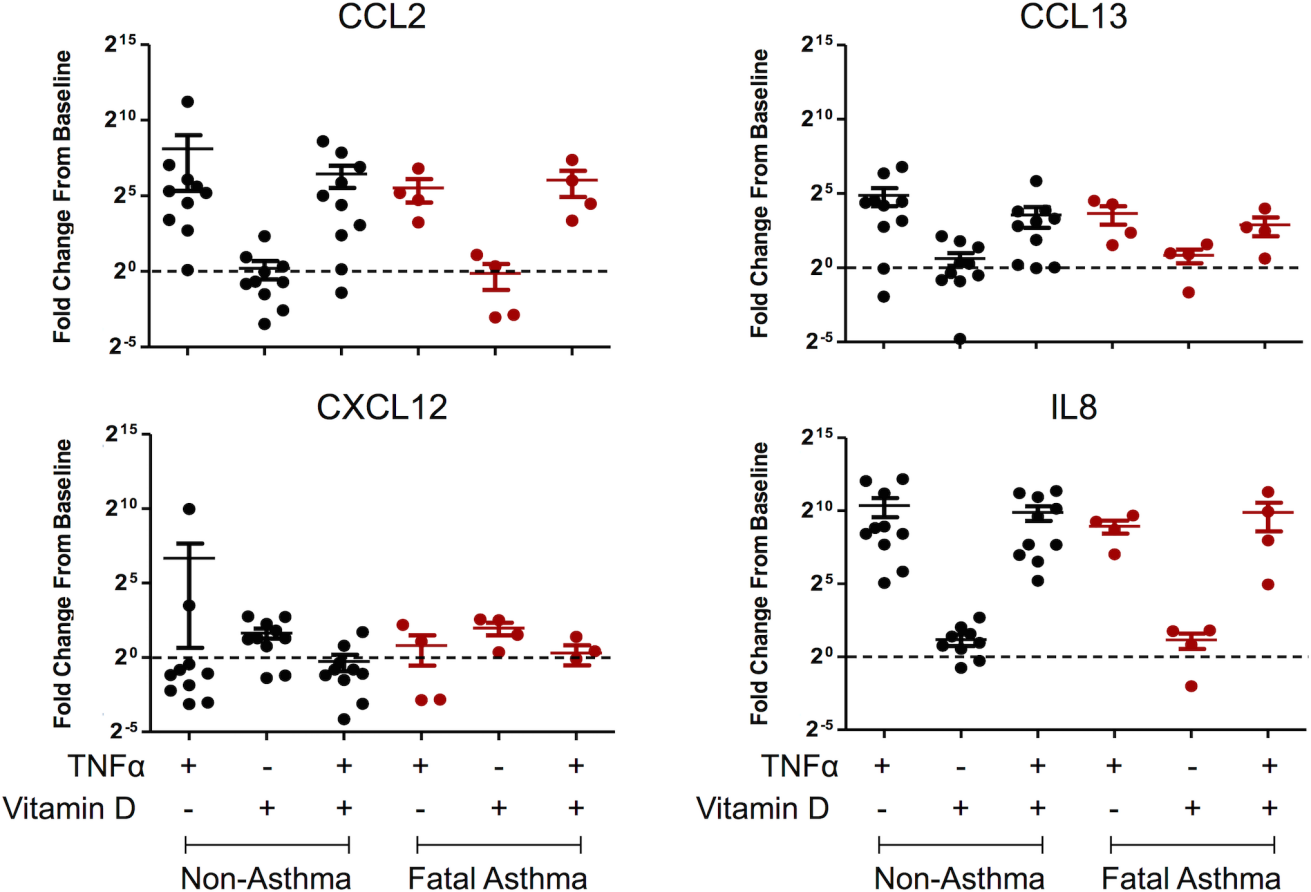
Vitamin D and TNFα Responsiveness of *CCL2*, *CCL13*, *CXCL12*, and *IL8* by qRT-PCR using the *GABARAP* housekeeping gene as reference.

Four genes (i.e. *CCL2*, *CCL13*, *CXCL12*, *IL8*) that (1) were members of the *GO*:*0008009~chemokine activity* ontological category, which was significantly over-represented among genes differentially expressed in response to vitamin D treatment in both fatal asthma- and non-asthma-derived ASM [S5 and S6 Tables], and (2) were differentially expressed in fatal asthma vs. non-asthma-derived ASM at baseline [S2 Table] were selected for detailed examination [Fig 1; Table 5]. Differential expression for these four genes at baseline and with vitamin D treatment was measured via qRT-PCR. No statistically significant differences in mRNA expression between fatal asthma- and non-asthma-derived ASM at baseline were found (data not shown). Fold changes for *CCL2* and *CXCL12* expression with vitamin D treatment vs. baseline were consistent with RNA-Seq results for most samples, while *CCL13* and *IL8* had consistent effects for only some samples [Fig 2]. We found that TNFα treatment induced *CCL2*, *CCL13*, and *IL8* mRNA expression in most fatal asthma- and non-asthma-derived ASM samples, but TNFα treatment did not induce *CXCL12* [Fig 2]. Vitamin D decreased the mean TNFα-induced *CCL2*, *CCL13*, and *IL8* mRNA expression in most ASM groups, but this change was not statistically significant or consistent across all samples [Fig 2].

**Table 5:** Differential expression results for all comparison groups corresponding to four cytokine genes that were selected for further study. Genes were selected based on being (1) members of the *GO*:*0008009~chemokine activity* ontological category, which was significantly over-represented among genes differentially expressed in response to vitamin D treatment in both fatal asthma- and non-asthma-derived ASM, that were (2) differentially expressed in fatal asthma-derived vs. non-asthma-derived ASM at baseline. FPKM = fragments per kilobase of transcript per million mapped reads.

						Fatal Asthma vs. Non-Asthma at Baseline		Non-Asthma Vitamin D vs. Baseline		Fatal Asthma Vitamin D vs. Baseline	
Gene	Locus	Mean FPKM Non-Asthma Baseline	Mean FPKM Fatal Asthma Baseline	Mean FPKM Non-Asthma Vitamin D	Mean FPKM Fatal Asthma Vitamin D	Log2[Fold Change]	Q-value	Log2[Fold Change]	Q-value	Log2[Fold Change]	Q-value
*CCL2*	chr17:32582295–32584220	166.2	300.3	51.1	65.1	0.85	1.9E-03	-1.70	1.9E-03	-2.21	1.9E-03
*CCL13*	chr17:32683470–32685629	1.31	5.62	0.42	1.49	2.10	1.9E-03	-1.63	2.9E-02	-1.91	6.3E-03
*CXCL12*	chr10:44865604–44880545	681.0	294.2	912.2	1009.5	-1.21	1.3E-02	0.42	0.61	1.78	1.9E-03
*IL8*	chr4:74606222–74609433	0.87	4.31	0.09	0.16	2.31	1.9E-03	-3.22	1.9E-03	-4.79	1.9E-03

### Characterization of Cytokine Secretion and Sensitivity to Vitamin D

**Fig 3:**
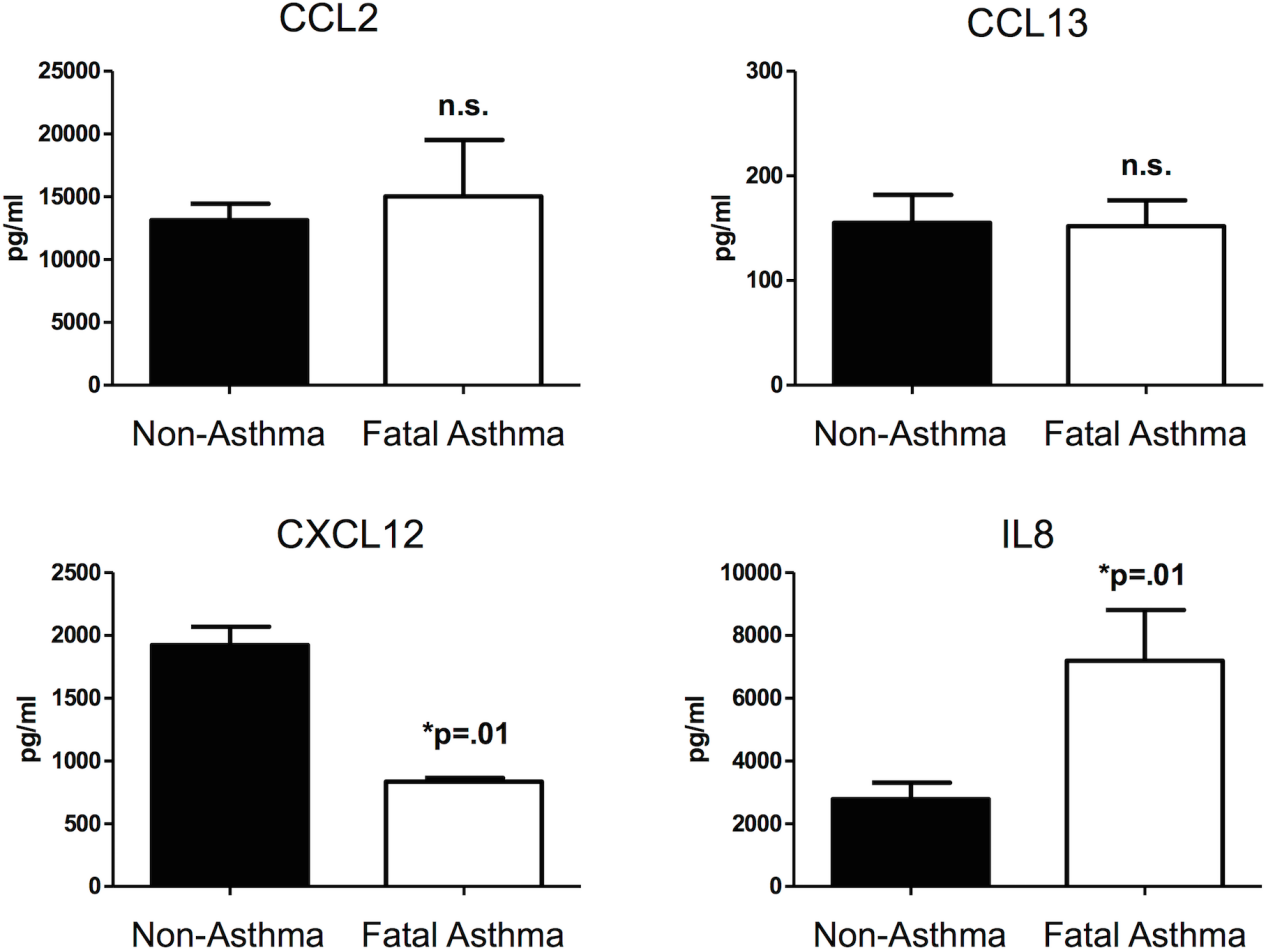
TNFα-induced cytokine levels in ASM. Cytokine levels in supernatant obtained from ASM derived from fatal asthma and non-asthma donors were determined by single ELISA. The data represent means ± standard error from ASM cells of 3 fatal asthma and 3 non-asthma donors for CCL2 and CXCL12; 5 fatal asthma and 6 non-asthma donors for CCL13; and 5 fatal asthma and 5 non-asthma donors for IL8 (numbers based on availability of cells used after RNA-Seq experiments). Each observation was performed in triplicate. Statistical significance was determined by Student’s one-tailed *t*-test with significance determined at p<0.05.

**Fig 4:**
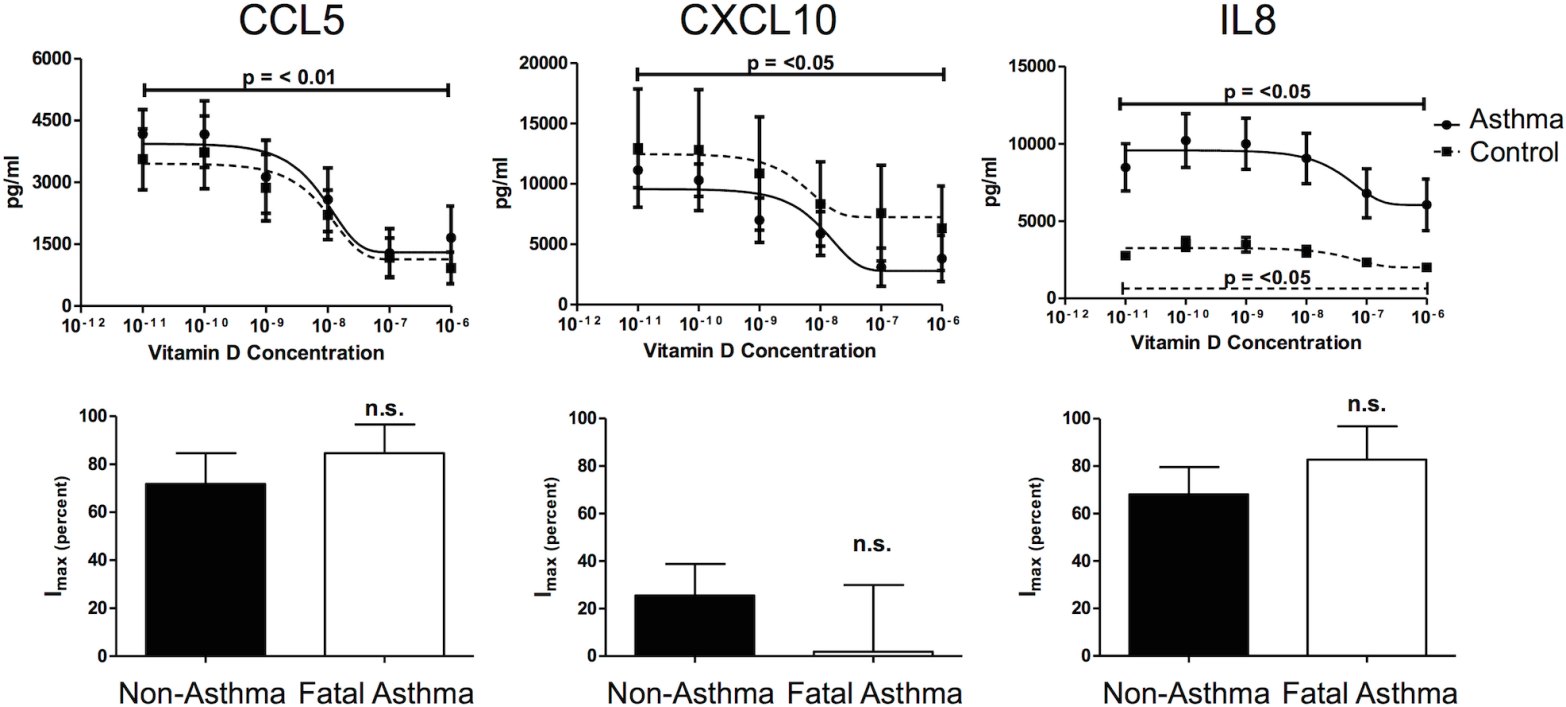
Vitamin D inhibited TNFα-induced cytokine expression. Although the amount of inhibition differed by cytokine, vitamin D inhibited three cytokine levels to a comparable degree in fatal asthma-derived vs. non-asthma-derived ASM, even for IL8, whose TNFα-induced baseline secretion was significantly higher in fatal asthma- vs. non-asthma-derived ASM. Data represent means ± standard error of the mean for ASM cells derived from 5 fatal asthma donors and 10 non-asthma donors. Each condition was measured in triplicate. Statistical significance was determined using Student’s two-tailed *t*-test with a p<0.05 threshold. Bottom panels compare baseline TNFα-induced cytokine levels to those obtained with a maximal inhibitory vitamin D concentration (I_max_).

Our RNA-Seq results suggested that mRNA for four cytokines were differentially expressed in ASM derived from fatal asthma vs. non-asthma donors at baseline and with vitamin D treatment. To address whether these differences in mRNA extended to changes in protein secretion, individual analyte ELISAs for the four selected cytokines (i.e. CCL2 (aka MCP-1), CCL13 (aka MCP-4), CXCL12 (aka SDF-1) and IL8) were performed. At baseline, CCL2 and CXCL12 were secreted differently from fatal asthma and non-asthma derived ASM in directions consistent with RNA-Seq results, but only those of CXCL12 were statistically significant [Fig 3]. IL8 secretion was not detected at baseline, while that of CCL13 was low and not different between fatal asthma and non-asthma derived ASM [Fig 3]. Fatal asthma-derived ASM secreted greater TNFα-induced IL8 and lower TNFα-induced CXCL12 as compared to non-asthma-derived ASM, while TNFα-induced CCL2, and CCL13 levels between fatal asthma- and non-asthma-derived ASM were not significantly different [Fig 3]. Overall, TNFα-induced secretion of CCL13 was low, while that of CCL2 and IL8 were high. Because IL8 was secreted at high levels that were significantly different between fatal asthma and non-asthma-derived ASM, we tested the dose-dependent effect of vitamin D on its TNFα-induced secretion. Additionally, CCL5 (aka RANTES), CXCL10 (aka IP-10) were also chosen for comparison experiments because these chemokines were known mediators in asthma that are differentially sensitive to vitamin D [11]. As shown in Fig 4, vitamin D inhibited three cytokine levels to a comparable degree in fatal asthma- vs. non-asthma-derived ASM, even for IL8, whose TNFα-induced baseline secretion was significantly higher in fatal asthma- vs. non-asthma-derived ASM. Although vitamin D was an effective inhibitor of TNFα-induced cytokine secretion, none of the TNFα-induced cytokine levels were completely abrogated. Further, the amount of TNFα-induced cytokine inhibition by vitamin D differed among cytokines, with IL8 levels being inhibited the least in fatal asthma- and non-asthma-derived ASM cells.

## Discussion

Previous studies have focused on understanding the potential therapeutic role of vitamin D in asthma because lower vitamin D levels are associated with increased asthma exacerbations and anti-inflammatory medication use, and decreased lung function [7,15]. A major mode of vitamin D (1,25(OH)_2_D_3_) action is to directly alter the expression of genes [16]. Understanding the role of vitamin D in modifying the ASM transcriptome of fatal asthma patients may thus provide insights into the mechanisms by which vitamin D influences severe asthma. Our RNA-Seq results found that a large number of genes are differentially expressed in the ASM of fatal asthma vs. non-asthma donors, and that the responsiveness to vitamin D treatment differs between these two groups. Vitamin D treatment of both fatal asthma- and non-asthma-derived ASM resulted in the differential expression of many cytokines and chemokines, but corresponding ontological categories were not found to be over-represented in the baseline comparison between fatal asthma- and non-asthma-derived ASM. Vitamin D receptor (*VDR*) mRNA expression levels were not different between fatal asthma- and non-asthma derived ASM [S3 Fig], suggesting that gene expression differences between these groups in response to vitamin D treatment did not simply reflect vitamin D sensitivity differences proportional to vitamin D receptor availability.

We chose to focus our functional studies on four cytokines that were differentially expressed between fatal asthma- and non-asthma-derived ASM and were also modified by vitamin D treatment in fatal asthma-derived ASM (i.e., *CCL2*, *CCL13*, *CXCL12*, *IL8*). While each of these genes was differentially expressed in fatal asthma- vs. non-asthma-derived ASM at baseline and with vitamin D treatment in fatal asthma-derived ASM, expression variability across samples was evident [Fig 1]. Subsequent qPCR results did not replicate RNA-Seq differential expression between fatal asthma- and non-asthma-derived ASM at baseline, and were only partly consistent with vitamin D effects: consistent with RNA-Seq results, *CCL2* had decreased and *CXCL12* had increased mRNA with vitamin D treatment for most samples, while *CCL13* and *IL8* manifested opposite mean effects [Fig 2]. These disparate findings could be due to inter-individual variability, unaccounted for experimental error by either RNA-Seq or qPCR, limitations in the methods used to analyze RNA-Seq data, and/or the intrinsic differences in the experimental techniques. In the case of *CCL13* and *IL8*, mRNA expression levels were low as evidenced by having most RNA-Seq FPKM values <10, which may have contributed toward inconsistency in measures between RNA-Seq and qPCR, as results from lowly expressed genes are known to be more error prone [30]. There is no consensus about the optimal way to analyze RNA-Seq data and considerable variability in results obtained based on platform and data analysis pipeline has been observed [31]. While RNA-Seq measures for each condition were normalized by taking some global measures into account (e.g., the total number of reads that mapped to the reference genome), qPCR measures were obtained in reference to a housekeeping gene only. We selected *GABARAP* as a housekeeping gene after finding that its coefficient of variation, 23.52%, was lowest among that of four genes (i.e. *ACTB*, *HPRT1*, *GABARAP*, *RPL19*) [S4 Fig]. Repetition of qPCR results using *ACTB* as a housekeeping gene found that similar trends emerged as when using *GABARAP* as reference, despite *GABARAP* having a greater coefficient of variation (47.92%) [S5 Fig]. We selected the housekeeping gene with lowest amount of variation to minimize the impact that its variability of expression would have on our ability to measure changes in the expression of other genes of interest. However, because a housekeeping gene with even greater coefficient of variability yielded similar results, the housekeeping gene choice may not have been the main source of inconsistency between RNA-Seq and qPCR results. Further sequencing and qPCR experiments using a greater number of individuals may clarify the source of inconsistency between techniques.

Comparison of RNA and protein expression for the four cytokines in Figs 1 and 3 shows (1) decreased CXCL12 mRNA, protein secretion at baseline, and TNFα-induced protein secretion in fatal asthma- vs. non-asthma-derived ASM, (2) increased CCL2 mRNA, protein secretion at baseline, and TNFα-induced protein secretion in fatal asthma- vs. non-asthma-derived ASM, although the protein secretion results are not statistically significant, (3) increased IL8 mRNA and TNFα-induced protein secretion in fatal asthma- vs. non-asthma-derived ASM, but undetectable levels of protein secretion at baseline, and (4) increased CCL13 mRNA in fatal asthma- vs. non-asthma-derived ASM, but unchanged protein secretion levels. Comparison of TNFα-induced mRNA and protein expression levels of for the four cytokines in Figs 2 and 3 shows that CCL2, CCL13, and IL8 had increased levels in both fatal asthma- and non-asthma-derived ASM, while CXCL12 had decreased levels in all but a few qPCR samples. Thus, consistency between mRNA and protein secretion levels is high for TNFα-induced changes, and present to varying degrees among the four cytokines for changes between fatal asthma- and non-asthma-derived ASM, with CXCL12 and IL8 showing the greatest consistency and CCL13 the weakest. While sometimes the levels of mRNA correlate with those of protein secretion, there are various stages between transcription and protein secretion that could bar a simple relationship, including protein translation, post-translational modifications that activate proteins and/or avoid degradation, and processes that successfully export protein from cells [32,33]. Based on our results, CCL2, CXCL12, and IL8 may have correlated mRNA and protein secretion levels that differ between fatal asthma- and non-asthma-derived ASM, but the difference in IL8 may only be noticeable with TNFα induction due to its low baseline levels. The mRNA differences of CCL13 between fatal asthma- and non-asthma-derived ASM did not extend to protein secretion levels. Further studies that address in more depth the mechanisms by which cytokine mRNA changes lead to protein level and secretion differences are needed to support our observed correlations for CCL2, CXCL12, and IL8.

The *CCL2*, *CCL13*, *CXCL12*, and *IL8* cytokine genes selected for experimental follow-up are known to be involved in asthma related processes. A previous study found that CCL2 (aka MCP1) concentration was elevated in primary ASM supernatant and blood of asthma patients, its sputum levels were increased in asthma patients with bronchial wall thickening, and chemotaxis assays suggested that it mediates fibrocyte migration toward ASM [34]. CCL2 has also been found to promote mast cell recruitment [35] and Th17 cell migration [36] to the lung in mouse models of asthma. CCL13 (aka MCP4) is a monocyte chemoattractant that has been proposed as a biomarker of asthma because its levels were higher in plasma of (1) asthma patients vs. non-asthma controls and (2) asthma patients with acute exacerbations vs. stable asthma [37], and its receptor, CCR3, has been shown to induce migration of ASM cells *in vitro* [38]. CXCL12, a chemotactic factor with pleiotropic effects that are modulated by cofactors, recruits cell types including leukocytes [39] and mast cells [40]. A potential asthma therapeutic based on blocking of CXCL12 has been shown to reduce airway eosinophil recruitment in an ovalbumin mouse model of asthma [41]. Further, airway mucosa from asthma patients had a greater number of CXCL12 (aka SDF1) positive cells than that from healthy subjects, and the number of CXCL12+ cells correlated with vascularity, suggesting that CXCL12 may play a role in airway remodeling [42]. Finally, IL8 is a neutrophil chemoattractant whose levels have been reported to be elevated in the sputum of asthma patients, suggesting that it may play a role in severe asthma that is characterized by neutrophilia [43,44]. Our RNA-Seq results showing that *CCL2*, *CCL13*, and *IL8* mRNA levels were elevated in fatal asthma-derived ASM are consistent with these previous studies showing elevated levels in ASM (for *CCL2*) and other tissues. Our RNA-Seq results for *CXCL12* showing decreased mRNA levels in fatal asthma have an opposite effect from that expected based on increased CXCL12+ cell types in airway mucosa, but a direct comparison of these studies’ results is limited because of the different experimental designs. Our work extends what is known about these four cytokines by suggesting a potential role of vitamin D in regulating their levels in ASM. However, dysregulation of these cytokines is a complex process with high variability across individuals, and further studies are required to better understand the mechanisms by which vitamin D may modulate their effects.

Changes in expression and secretion of individual genes in ASM cells in response to vitamin D treatment have been studied previously. For example, in a study of the inhibitory properties of vitamin D on ASM proliferation, *MMP9* and *ADAM33* were selected as candidate genes via which vitamin D might affect ASM hyperplasia and both were differentially expressed with vitamin D treatment [14]. In our results, these genes were not differentially expressed in either of the three comparisons made, but other genes representing the extracellular matrix were significantly represented, including other matrix metalloproteinases (e.g., *MMP1*, *MMP12*) and ADAMTS proteases (e.g., *ADAMTS10*, *ADAMTS15*) [S5 and S6 Tables]. A previous study of the role of vitamin D in ASM inflammation found that vitamin D decreased expression of *KPNA4* (a.k.a. importin α3), a mediator of NFκB-induced inflammation, by TNFα and IL1β in the ASM [12], but in our study, *KPNA4* mRNA was not differentially expressed in any of the comparison groups. Another study found that TNFα/IFNγ-induced secretion of *CX3CL1* was significantly decreased by vitamin D treatment [11]. Consistent with this result, we found that *CX3CL1* expression was decreased in non-asthma-derived ASM treated with vitamin D vs. at baseline (q-value 0.011; log2 fold-change 2.3), but the findings did not hold in fatal asthma-derived ASM. TNFα-induced CCL5 (aka RANTES) and CXCL10 (aka IP-10) chemokine secretion were decreased in ASM cells treated with vitamin D [11], but we did not observe any mRNA expression changes for *CCL5* or *CXCL10*. These differences between protein secretion and transcript expression suggest that some changes in cytokine secretion levels are not transcriptionally mediated, or that stimulation with an appropriate cytokine is necessary to observe the changes.

On a transcriptomic level, the effects of vitamin D on ASM gene expression were measured in a previous microarray study that used cells from a single non-asthmatic male donor stimulated with 100nM 1α,25(OH)_2_D_3_ for 24 hours [17]. Our results are consistent with the results reported in that paper. For example, *CDC42EP3*, *CEBPB*, *G6PD*, *HSD11B1*, *IL6*, and *ITPR1* were highlighted by this previous study as asthma-relevant genes that were differentially expressed in response to vitamin D treatment, and each of these is among the 394 genes with lowest q-value of 1.92E-03 in our baseline vs. vitamin D treated non-asthma-derived ASM comparison [S7 Table]. A subset of these genes (i.e., *CDC42EP3*, *ITPR1*, *G6PD*) was differentially expressed in the fatal asthma- vs. non-asthma-derived ASM at baseline comparison, while a different subset (i.e., *CEBPB*, *CDC42EP3*, *ITPR1*, and *HSD11B1*) was differentially expressed in fatal asthma-derived ASM when stimulated with vitamin D vs. left untreated [S7 Table]. The differences in significant genes among comparison groups may reflect biological differences in vitamin D responsiveness between fatal asthma- and non-asthma-derived ASM cells. Thus, an important strength of our study compared to previous reports is the inclusion of ASM cells from diseased individuals, which may aid in identifying transcriptomic differences that are most relevant to asthma.

The ontological categories represented by differentially expressed genes in S1, S3, and S4 Tables are consistent with biological processes known to be important in severe asthma. The extracellular matrix is important in ASM remodeling [45], steroid resistance and oxidative stress directly contribute to severe asthma [6], and differences in lung development and response to wounding may contribute to intrinsic differences in ASM that alter asthma severity. Two categories that were not over-represented by differentially expressed genes but might be expected are those related to smooth muscle contraction and smooth muscle proliferation since the ASM regulates airway contractility and ASM proliferation contributes to increased ASM mass [45]. Although statistical significance was not achieved, categories related to these processes characteristic of smooth muscle function were represented by genes significantly differentially expressed in some comparisons. Specifically, *GO*:*0048661~positive regulation of smooth muscle cell proliferation* included seven differentially expressed genes (i.e. *PRKCA*, *IL6*, *S1PR1*, *PTGS2*, *VEGFA*, *IGF1*, *ITGA2*) in non-asthma-derived ASM and six differentially expressed genes (i.e. *CDH13*, *VEGFA*, *TGM2*, *IGF1*, *ITGA2*, *STAT1*) in fatal asthma-derived ASM, with Benjamini-Hochberg corrected p-values of 0.066 and 0.14, respectively. This category was absent in the fatal asthma- vs. non-asthma-derived ASM baseline comparison. The category *GO*:*0006939~smooth muscle contraction* included four differentially expressed genes (i.e., *ACTG2*, *EDNRB*, *AGT*, *MYH11*) in the fatal asthma- vs. non-asthma-derived ASM comparison, six differentially expressed genes (i.e., *KCNMA1*, *EDNRB*, *CACNA1G*, *BDKRB2*, *CACNA1C*, *GDNF*) in the non-asthma-derived ASM baseline vs. vitamin D treatment comparison, and four differentially expressed genes (i.e., *KCNMA1*, *EDNRA*, *ACTG2*, *AGT*) in the fatal asthma-derived ASM baseline vs. vitamin D treatment comparison, with Benjamini-Hochberg corrected p-values of 0.80, 0.22 and 0.69, respectively. Thus, while groups of genes of some relevant ontological categories may not have been differentially expressed, transcriptional changes of individual genes that influence ASM contractility and proliferation were present.

A limitation of our study is that we used ASM cells from white donors to study responsiveness to vitamin D. Dose response rates [46] and vitamin D protein binding levels [47] by race, suggesting that gene expression differences in response to vitamin D treatment also differ by race. Because vitamin D deficiency [48] and asthma prevalence, hospitalizations, and death rates [49] are known to be higher among black than white patients, future studies of the effect of vitamin D on the ASM transcriptome that include individuals of diverse racial/ethnic backgrounds may reveal additional gene candidates and/or biological pathways that shed light on asthma disparities. Another limitation is that our RNA-Seq analyses were limited to the hg19 RefSeq annotation files downloaded from Illumina’s iGenomes project. Thus, we did not characterize the expression of long-non-coding RNA or mRNA transcript isoforms that were not part of the reference file used. We opted for use of a well-annotated reference file for our investigation of the ASM transcriptome to reduce the number of false-positive results. Future studies with more comprehensive annotation files, a greater number of individuals, and/or greater sequencing depth will yield additional insight into the ASM transcriptome.

In summary, we found that 838 genes were differentially expressed in fatal asthma- vs. non-asthma-derived ASM at baseline, and vitamin D treatment vs. baseline conditions resulted in differential expression of 867 and 711 genes in non-asthma- and fatal asthma-derived ASM, respectively. Functional categories that were represented in all groups include extracellular matrix, response to steroid hormone stimulus, and response to wounding. Genes differentially expressed by vitamin D also represented cytokine and chemokine activity categories. Further experiments conducted for four cytokines (i.e. CCL2, CCL13, CXCL12, IL8) selected from among the top RNA-Seq results found that vitamin D inhibited TNFα-induced IL8 protein secretion levels to a comparable degree in fatal asthma- and non-asthma-derived ASM even though IL8 had significantly higher baseline levels in fatal asthma-derived ASM. Our results provide transcriptome data to further explore the functional differences in the ASM of fatal asthma vs. non-asthma donors, as well as differences in their vitamin D responsiveness.

## Materials and Methods

### Ethics Statement

Lung tissue was obtained from the National Disease Resource Interchange (NDRI) and its use approved by the University of Pennsylvania Institutional Review Board; use of the cells does not constitute human subjects research since all donor tissue is harvested anonymously and de-identified.

### ASM Cell Culture and Vitamin D Treatment for RNA-Seq Experiment

Primary ASM cells were isolated from white donors, six who died of fatal asthma and ten with no chronic illness or medication use. ASM cell cultivation was described previously [50,51]. ASM cells up to passage 4 maintained in Ham's F12 medium supplemented with 10% FBS, CaCl_2_, buffered with HEPES, penicillin/streptomycin, primocin, and additional L-glutamine were used in all experiments. The F12 medium was used for culture because it provides Ca^2+^ levels that are consistent with seeing contractility of muscles in that media. Following 48 hours of serum deprivation, cells from each donor were treated with 100 nM vitamin D (1,25(OH)_2_D_3_, Cayman Chemical Company, Ann Arbor, MI) or control vehicle for 18 h. This treatment protocol is biologically relevant (approximately 80μg/dL of vitamin D), maximally induces expression of *CYP24A1*, a well-known metabolizer of vitamin D, and inhibits cytokine-induced cytokine and chemokine expression in ASM [11,13].

### RNA-Seq Library Construction and Sequencing

Total RNA was extracted from cells using the miRNAeasy mini kit (Qiagen Sciences, Inc., Germantown, MD). Approximately 1 μg of RNA from each sample was used to generate RNA-Seq cDNA libraries for sequencing using the TruSeq RNA Sample Prep Kit v2 (Illumina, Inc., San Diego, CA). Sample preparation followed the manufacturer’s protocol with a workflow that included isolation of poly-adenylated RNA molecules using poly-T oligo-attached magnetic beads, enzymatic RNA fragmentation, cDNA synthesis, ligation of bar-coded adapters, and PCR amplification. Ambion External RNA Controls Consortium (ERCC) RNA Spike-In Control Mix 1 (Life Technologies Corporation, Carlsbad, CA) was added to the samples. The amplified cDNA fragments were analyzed using the 2100 Bioanalyzer (Agilent Technologies, Inc., Santa Clara, CA) to determine fragment quality and size. Library concentrations were determined by Qubit Fluorometric Quantitation (Life Technologies Corporation, Carlsbad, CA). Sequencing of 75 bp paired-end reads was performed with an Illumina HiSeq 2000 instrument at Partners Personalized Medicine (Boston, MA).

### RNA-Seq Data Analysis

Preliminary processing of raw reads was performed using Casava 1.8 (Illumina, Inc., San Diego, CA). Subsequently, Taffeta scripts (https://github.com/blancahimes/taffeta) were used to analyze RNA-Seq data, which included trimming of adapters using trimmomatic (v.0.22) [52] and using FastQC [53] (v.0.10.0) to obtain overall QC metrics. Trimmed reads for each sample were aligned to the reference hg19 genome and known ERCC transcripts using TopHat [54] (v.2.0.8), while constraining mapped reads to be within reference hg19 or ERCC transcripts. Additional QC parameters were obtained to assess whether reads were appropriately mapped. Bamtools (v.1.0.2) [55] was used to count/summarize the number of mapped reads, including junction spanning reads. The Picard Tools (v.1.58; http://picard.sourceforge.net) RnaSeqMetrics function was used to compute the number of bases assigned to various classes of RNA, according to the hg19 refFlat file available as a UCSC Genome Table. For each sample, Cufflinks [18] (v.2.1.1) was used to quantify ERCC Spike-In and hg19 transcripts based on reads that mapped to the provided hg19 and ERCC reference files. ERCC Spike-ins dose response curves (i.e. plots of ERCC transcript FPKM vs. ERCC transcript molecules) were created following the manufacturer’s protocol [56]. Raw read plots were created by displaying bigwig files for each sample in the UCSC Genome Browser. The RNA-Seq data is available at the Gene Expression Omnibus Web site (http://www.ncbi.nlm.nih.gov/geo/) under accession GSE58434.

### RNA-Seq Quality Control

Initially, 36 RNA samples from 18 vitamin D treated and untreated pairs were obtained. One pair of RNA samples was excluded from sequencing preparation due to failed RIN scores, indicating very low RNA quality. Two samples with evidence of poor quality RNA-Seq library (based on low percentage of junction spanning reads, percentage of mRNA bases and mean insert size [S8 Table]) and the two paired samples matching them, were also excluded, leaving a total of 30 samples selected for differential expression analysis. Such samples corresponded to 5 fatal asthma and 10 non-asthma-derived donors with vitamin D or control vehicle treatment [Table 1]. For these 30 samples, we obtained an average of 93.2 million raw sequencing reads per sample (range 73.0–121.7 million reads per sample). Of these reads, an average of 89.1% were aligned to hg19 genome reference files downloaded from Illumina’s iGenomes project (range 85.9%-91.6%), and an average of 29.1% of the mapped reads spanned junctions (range 23.2%-31.4%). An average of 92.7% of bases in mapped reads corresponded to mRNA (range 73.6%-96.2%). ERCC spike-in dose response plots, which ideally would have slope and R^2^ equal to 1.0, had slope and R^2^ > = 0.91. Based on these and other quality control (QC) summary metrics, the sequencing and alignment results for each of the 30 samples were deemed of sufficiently high quality to include in differential expression analyses [S8 Table]. Quantification of transcript and gene expression levels, according to hg19 RefSeq annotation files from Illumina’s iGenomes Project, was performed using Cufflinks. As positive controls of gene expression, fragments per kilobase of transcript per million mapped reads (FPKM) values for three housekeeping genes (i.e., *B2M*, *GABARAP*, *RPL19*), one well known metabolizer of vitamin D (i.e., the cytochrome P450 gene *CYP24A1* [57]), and the vitamin D receptor (i.e., *VDR*) were obtained. Each housekeeping gene had high FPKM values that did not differ significantly by treatment status, *CYP24A1*’s barely detectable baseline levels greatly increased in response to vitamin D treatment [S2 Fig], and *VDR* had a trend toward increase in response to vitamin D treatment but did not differ significantly by treatment or fatal asthma vs. non-asthma status [S3 Fig].

Differential expression of genes and transcripts was obtained using Cuffdiff [18] (v.2.1.1) with the quantified transcripts computed by Cufflinks (v.2.1.1), while applying bias correction for all samples. The CummeRbund [58] R package (v.2.0.0) was used to measure significance of differentially expressed genes and create plots of the results. The reported q-values are false-discovery rate adjusted p-values according to the implementation in Cuffdiff and CummeRbund that take into account the large number of comparisons made. Comparison groups of interest were: 1) fatal asthma- vs. non-asthma-derived ASM samples at baseline; 2) fatal asthma-derived ASM samples treated with vitamin D vs. left untreated; 3) non-asthma-derived ASM samples treated with vitamin D vs. left untreated. The NIH Database for Annotation, Visualization and Integrated Discovery (DAVID) was used to perform gene functional annotation clustering using Homo Sapiens as background, and default options and annotation categories (Disease: OMIM_DISEASE; Functional Categories: COG_ONTOLOGY, SP_PIR_KEYWORDS, UP_SEQ_FEATURE; Gene_Ontology: GOTERM_BP_FAT, GOTERM_CC_FAT, GOTERM_MF_FAT; Pathway: BBID, BIOCARTA, KEGG_PATHWAY; Protein_Domains: INTERPRO, PIR_SUPERFAMILY, SMART) [29].

### Quantitative Real-Time PCR (qRT-PCR) Analysis

Following 48 hours of serum deprivation, ASM cells from 4 fatal and 10 non-asthma-derived donors (all used for RNA-Seq) were treated with 100nM vitamin D or vehicle control for 1h, followed by stimulation with 10ng/mL TNFα or vehicle control for 18h. Total RNA was isolated from cells by using QIAshredder and RNeasy kits (Qiagen Sciences, Inc., Germantown, MD). Oligo(dT)-primed cDNA was prepared from 500 ng of total RNA by using SuperScript III First-strand Synthesis System (Invitrogen, Life Technologies, Grand Island, NY). qRT-PCR was set up in the presence of 0.5 μM primers for *CCL2*, *CCL13*, *CXCL12*, and *IL8* by using QuantiTect SYBR Green PCR kit (Qiagen Sciences, Inc., Germantown, MD). qRT-PCR was performed on an Eppendorf Mastercycler ep Realplex^2^ real time PCR machine (Eppendorf, Hauppauge, NY). *GABARAP* was used as an internal control for data normalization as it was the housekeeping gene with the lowest amount of variation among six ASM samples (3 fatal asthma-derived, 3 non-asthma-derived) for four housekeeping genes tested (i.e. *ACTB*, *HPRT1*, *GABARAP*, *RPL19*) [S4 Fig]. Additionally, we repeated qRT-PCR using *ACTB* as a housekeeping gene to measure the extent of result variability given the housekeeping gene choice [S5 Fig]. Fold increase of expression was calculated using the delta-delta CT method.

### Cytokine ELISAs

To test whether some of our RNA-Seq results extended to protein secretion levels, ELISAs were performed for four cytokines. Human ASM cells were grown to confluence and then serum deprived for 48 h. Monolayers were stimulated with 10 ng/ml TNFα (Roche Diagnostics Corporation, Indianapolis, IN), in the presence and absence of 100 nM vitamin D, or vehicle control. After an 18h incubation, supernatants were harvested and cytokine or chemokine levels measured by single analyte ELISAs (R&D Systems, Minneapolis, MN). Comparisons were made of baseline and TNFα-induced chemokine and cytokine levels in fatal asthma-derived ASM and those derived from non-asthma age- and gender-matched donors. Analyses were performed using ASM cells from 3 fatal asthma and 3 non-asthma donors for CCL2 and CXCL12; 5 fatal asthma and 6 non-asthma donors for CCL13; and 5 fatal asthma and 5 non-asthma donors for IL8 based on availability of cells used after RNA-Seq experiments. Each condition was performed in triplicate. Data was analyzed as means ± standard error of the means and statistics determined significance using one-tailed student’s t test.

## Supporting Information

S1 Fig RNA-Seq profiling of ASM cells. Volcano plots of overall gene-based differential expression results for A) fatal asthma- vs. non-asthma-derived ASM at baseline, B) non-asthma-derived ASM at baseline vs. when treated with vitamin D, C) fatal asthma-derived ASM at baseline vs. when treated with vitamin D. The y-axis corresponds to the negative log (base 10) of P-values while the x-axis corresponds to the negative log (base 2) of the fold change for difference in expression between categories. Differentially expressed genes according to an adjusted p-value <0.05 are colored in red.
(TIFF)

S2 Fig RNA-Seq results expressed as FPKM for three housekeeping genes (i.e., *B2M*, *GABARAP*, *RPL19*) and one well known metabolizer of vitamin D (i.e., the cytochrome P450 gene *CYP24A1*) by condition status show (1) high levels of expression for each housekeeping gene that did not significantly differ by condition status, and (2) induction of *CYP24A1* with vitamin D treatment in both fatal asthma- and non-asthma-derived ASM cells. (TIFF)

S3 Fig RNA-Seq results expressed as FPKM for the vitamin D receptor gene (i.e., *VDR*) by condition status show that its mRNA levels did not significantly differ by condition status. (TIFF)

S4 Fig Variation in 2^[-C_t_] values for four housekeeping genes (i.e., *ACTB*, *HPRT1*, *GABARAP*, *RPL19*) among six ASM samples (3 fatal asthma-derived, 3 non-asthma-derived). *GABARAP* was selected as a reference housekeeping gene for further qPCR experiments.
(TIFF)

S5 Fig Vitamin D and TNFα Responsiveness of *CCL2*, *CCL13*, *CXCL12*, and *IL8* by qRT-PCR using *ACTB* as a housekeeping reference gene shows similar differential expression results as those in Fig 2. (TIFF)

S1 Table Functional annotation clusters obtained with the NIH DAVID tool using all differentially expressed genes in fatal asthma- vs. non-asthma-derived ASM at baseline. Clusters with enrichment scores >1.5 are shown. Individual P-values listed correspond to EASE Scores, or modified Fisher Exact P-Values computed by DAVID.
(DOCX)

S2 Table Individual annotation categories for top 838 fatal asthma- vs. non-asthma-derived ASM differentially expressed genes obtained from NIH DAVID cluster analysis tool. Categories selected were from clusters with enrichment scores >2.50 and with individual Benjamini-Hochberg corrected p-values <0.05 that correspond to known asthma-related structures and processes, plus two categories that met these criteria in the vitamin D treated cells vs. those at baseline. Genes listed were the differentially expressed ones for the corresponding category.
(DOCX)

S3 Table Functional annotation clusters obtained with the NIH DAVID tool using all differentially expressed genes in non-asthma-derived ASM at baseline vs. when treated with vitamin D. Clusters with enrichment scores >1.5 are shown. Individual P-values listed correspond to EASE Scores, or modified Fisher Exact P-Values computed by DAVID.
(DOCX)

S4 Table Functional annotation clusters obtained with the NIH DAVID tool using all differentially expressed genes in fatal asthma-derived ASM at baseline vs. when treated with vitamin D. Clusters with enrichment scores >1.5 are shown. Individual P-values listed correspond to EASE Scores, or modified Fisher Exact P-Values computed by DAVID.
(DOCX)

S5 Table Individual annotation categories for top 867 non-asthma-derived ASM at baseline vs. with vitamin D treatment differentially expressed genes obtained from NIH DAVID cluster analysis tool. Categories selected were from clusters with enrichment scores >2.50 and with individual Benjamini-Hochberg corrected p-values <0.05 that correspond to known asthma-related structures and processes, plus categories that met these criteria in Table 3. Genes listed were the differentially expressed ones for the corresponding category.
(DOCX)

S6 Table Individual annotation categories for top 711 fatal asthma-derived ASM at baseline vs. with vitamin D treatment differentially expressed genes obtained from NIH DAVID cluster analysis tool. Categories selected were from clusters with enrichment scores >2.50 and with individual Benjamini-Hochberg corrected p-values <0.05 that correspond to known asthma-related structures and processes, plus categories that met these criteria in Table 3. Genes listed were the differentially expressed ones for the corresponding category.
(DOCX)

S7 Table Differential expression results for all comparison groups of asthma-related genes that were differentially expressed in ASM cells from a single male donor treated with vitamin D highlighted in previous work by Bosse, Y *et al* [17]. (DOCX)

S8 Table RNA-Seq Metrics Computed for Quality Control. The table contains the number of raw reads for the paired-end samples, the percentage of mapped reads among the total number of raw reads, the percentage of junction spanning reads among the mapped reads, the percentage of mapped bases that mapped to mRNA, the mean insert size of mapped reads, and the slope and R^2^ values of ERCC spike-in dose response curves. Samples that were not included in differential expression analyses are highlighted in grey. The two samples marked by an asterisk (*) were dropped because of low percentage of junction spanning reads and mRNA bases, and mean insert size. The other three samples highlighted in grey were dropped because their pairs were not successfully sequenced.
(DOCX)
