# Supplementary figures and images for: Vitamin D Modulates Expression of the Airway Smooth Muscle Transcriptome in Fatal Asthma
Source: PLoS One. 2015 Jul 24;10(7):e0134057. doi: 10.1371/journal.pone.0134057 (PMC4514847; doi:10.1371/journal.pone.0134057)

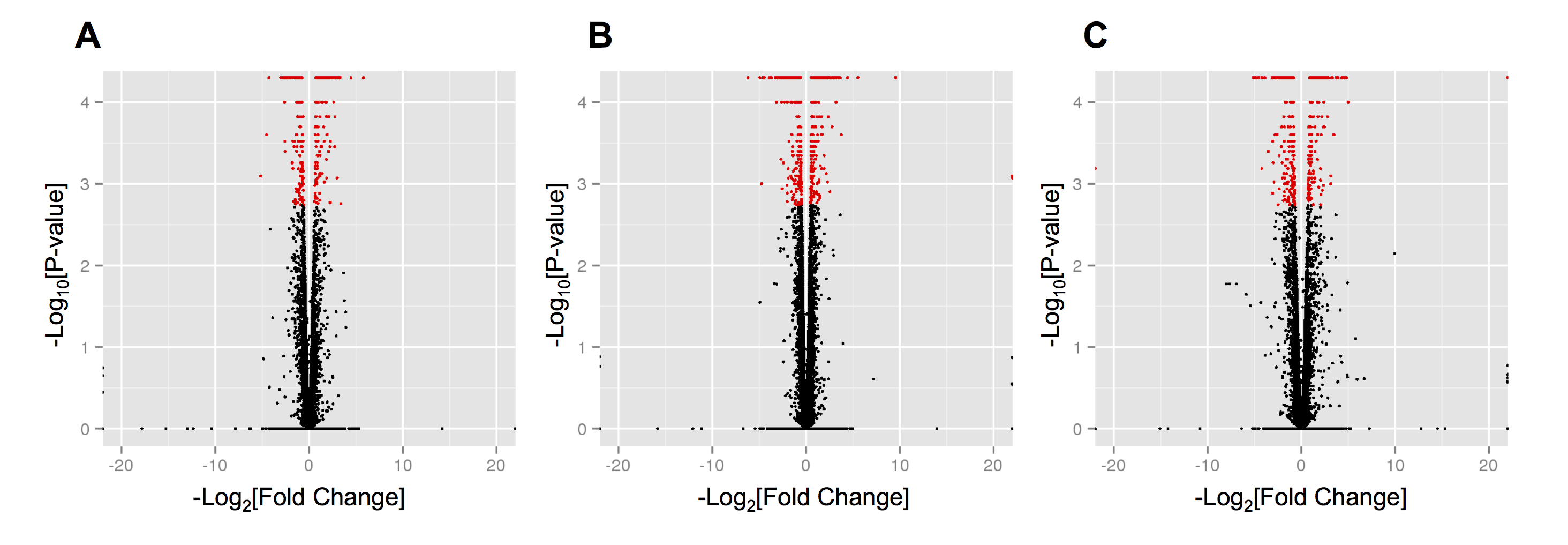

Supplement: S1 Fig — Volcano plots of overall gene-based differential expression results for A) fatal asthma- vs. non-asthma-derived ASM at baseline, B) non-asthma-derived ASM at baseline vs. when treated with vitamin D, C) fatal asthma-derived ASM at baseline vs. when treated with vitamin D. The y-axis corresponds to the negative log (base 10) of P-values while the x-axis corresponds to the negative log (base 2) of the fold change for difference in expression between categories. Differentially expressed genes according to an adjusted p-value <0.05 are colored in red. (TIFF) [file pone.0134057.s001.tiff]

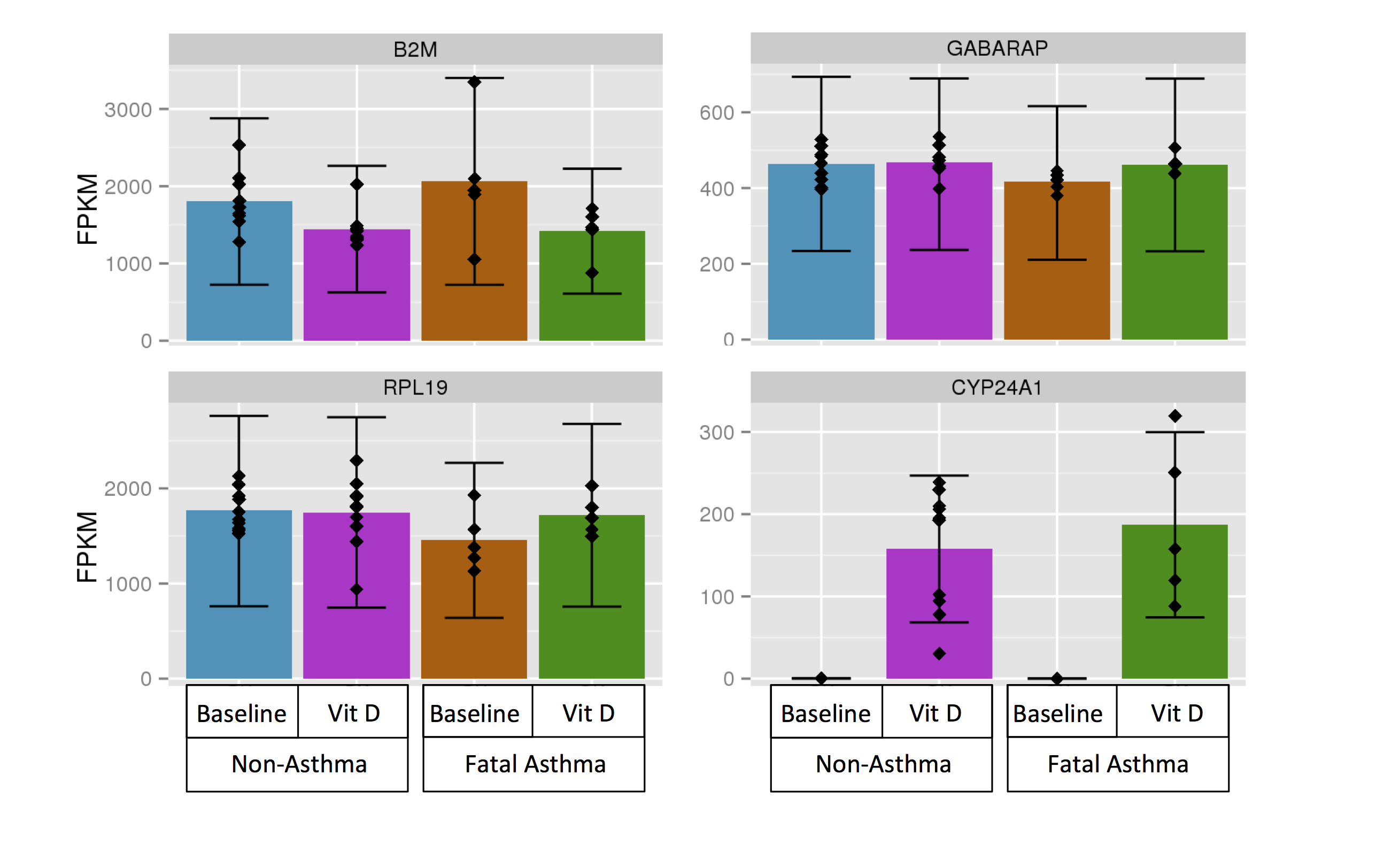

Supplement: S2 Fig — (TIFF) [file pone.0134057.s002.tiff]

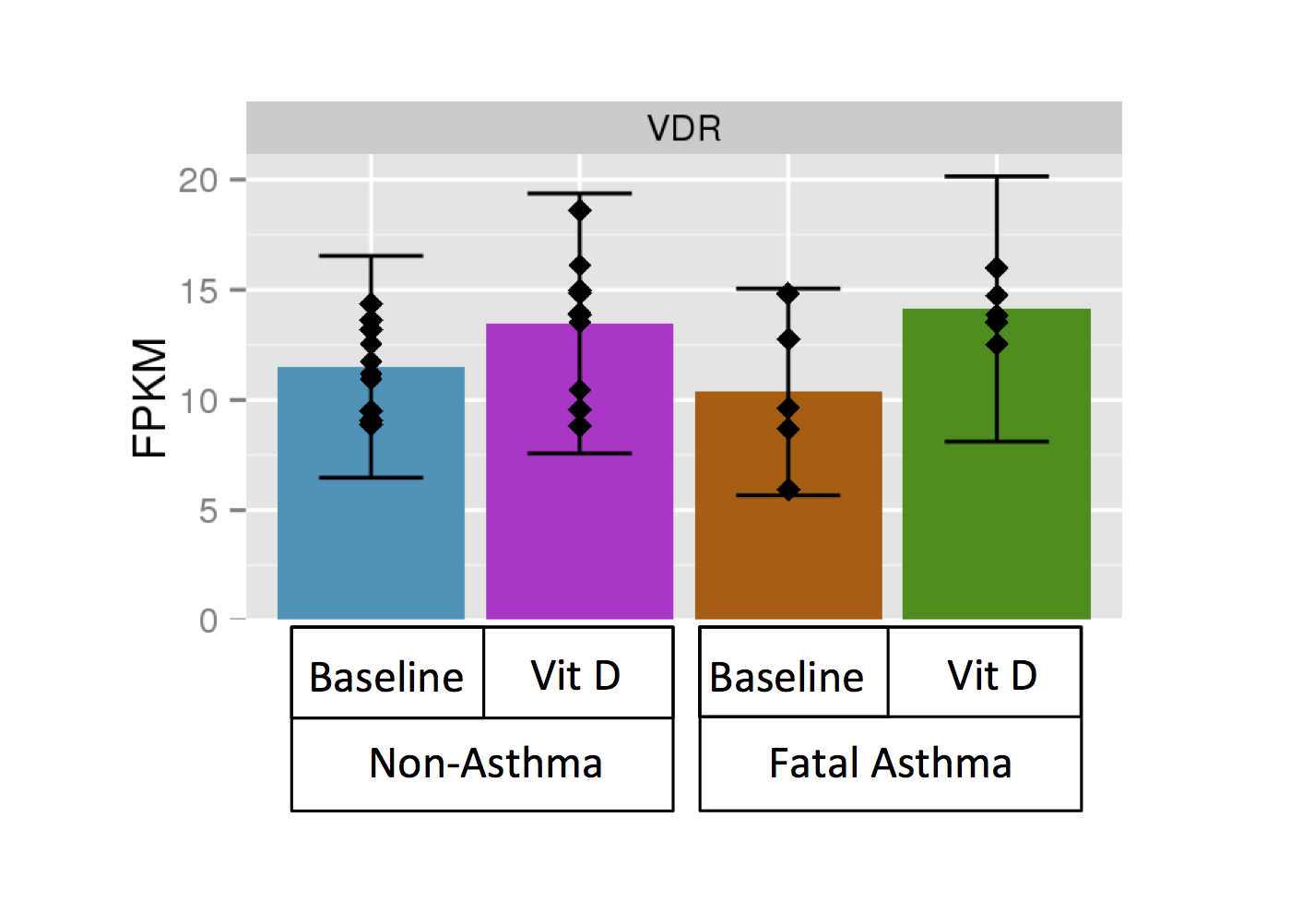

Supplement: S3 Fig — (TIFF) [file pone.0134057.s003.tiff]

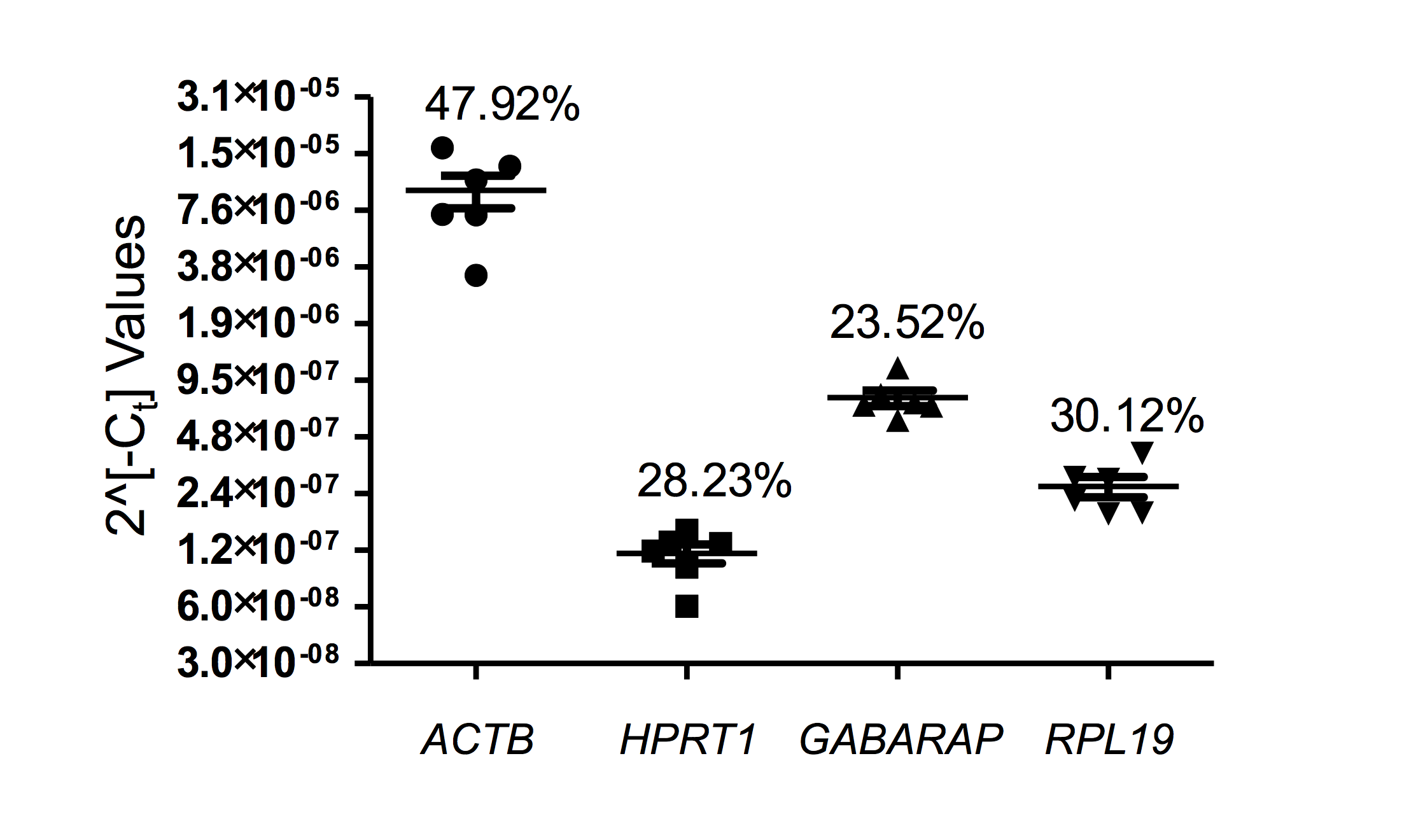

Supplement: S4 Fig — GABARAP was selected as a reference housekeeping gene for further qPCR experiments. (TIFF) [file pone.0134057.s004.tiff]

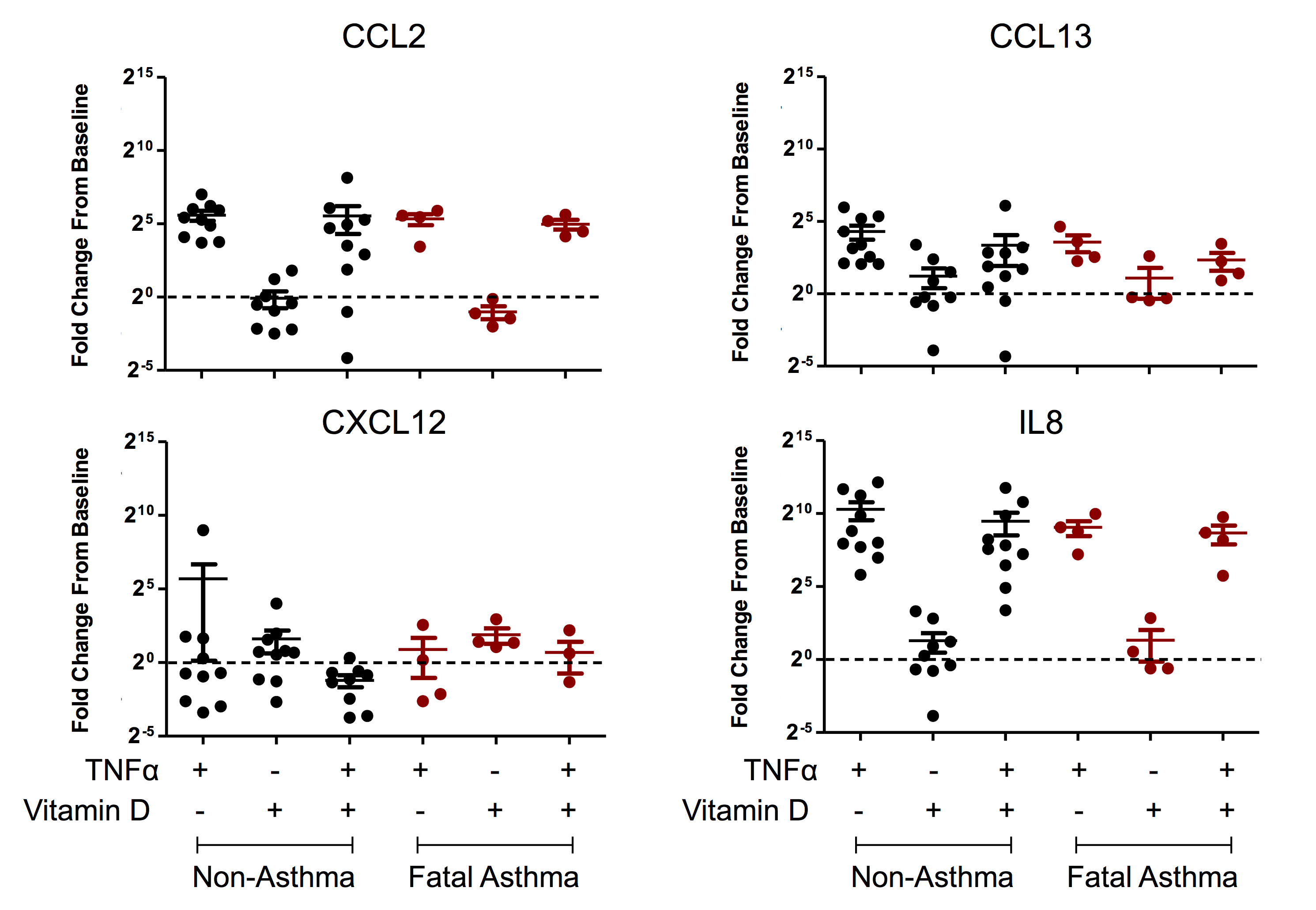

Supplement: S5 Fig — (TIFF) [file pone.0134057.s005.tiff]
