# Supplementary material for: Vitamin D Modulates Expression of the Airway Smooth Muscle Transcriptome in Fatal Asthma
Source: PLoS One. 2015 Jul 24;10(7):e0134057. doi: 10.1371/journal.pone.0134057 (PMC4514847; doi:10.1371/journal.pone.0134057)
Supplement: S1 Table — Clusters with enrichment scores >1.5 are shown. Individual P-values listed correspond to EASE Scores, or modified Fisher Exact P-Values computed by DAVID. (DOCX) [file pone.0134057.s006.docx]

| Annotation Cluster 1 | Enrichment Score: 29.08 |  |  |  |
| --- | --- | --- | --- | --- |
| Category | Term | Gene Count | P-Value | Benjamini-Hochberg P-value |
| SP_PIR_KEYWORDS | glycoprotein | 351 | 1.2E-43 | 6.9E-41 |
| UP_SEQ_FEATURE | glycosylation site:N-linked (GlcNAc...) | 331 | 1.8E-38 | 4.3E-35 |
| SP_PIR_KEYWORDS | signal | 278 | 2.7E-36 | 7.5E-34 |
| UP_SEQ_FEATURE | signal peptide | 278 | 8.3E-36 | 9.7E-33 |
| SP_PIR_KEYWORDS | Secreted | 164 | 9.7E-26 | 1.8E-23 |
| SP_PIR_KEYWORDS | disulfide bond | 221 | 1.2E-20 | 1.8E-18 |
| UP_SEQ_FEATURE | disulfide bond | 212 | 4.0E-19 | 3.2E-16 |
| GOTERM_CC_FAT | GO:0005576~extracellular region | 187 | 1.0E-18 | 2.0E-16 |
|  |  |  |  |  |
| Annotation Cluster 2 | Enrichment Score: 14.66 |  |  |  |
| Category | Term | Gene Count | P-Value | Benjamini-Hochberg P-value |
| GOTERM_CC_FAT | GO:0044421~extracellular region part | 115 | 5.9E-19 | 2.3E-16 |
| GOTERM_CC_FAT | GO:0031012~extracellular matrix | 58 | 9.0E-16 | 1.2E-13 |
| GOTERM_CC_FAT | GO:0005578~proteinaceous extracellular matrix | 54 | 8.7E-15 | 8.5E-13 |
| SP_PIR_KEYWORDS | extracellular matrix | 38 | 5.0E-12 | 5.6E-10 |
|  |  |  |  |  |
| Annotation Cluster 3 | Enrichment Score: 6.97 |  |  |  |
| Category | Term | Gene Count | P-Value | Benjamini-Hochberg P-value |
| GOTERM_BP_FAT | GO:0007155~cell adhesion | 71 | 1.6E-09 | 4.7E-06 |
| GOTERM_BP_FAT | GO:0022610~biological adhesion | 71 | 1.6E-09 | 2.4E-06 |
| SP_PIR_KEYWORDS | cell adhesion | 45 | 1.8E-08 | 1.3E-06 |
| GOTERM_BP_FAT | GO:0016337~cell-cell adhesion | 25 | 2.9E-03 | 9.5E-02 |
|  |  |  |  |  |
| Annotation Cluster 4 | Enrichment Score: 6.65 |  |  |  |
| Category | Term | Gene Count | P-Value | Benjamini-Hochberg P-value |
| GOTERM_CC_FAT | GO:0031226~intrinsic to plasma membrane | 105 | 2.6E-08 | 1.7E-06 |
| GOTERM_CC_FAT | GO:0005887~integral to plasma membrane | 102 | 6.1E-08 | 3.0E-06 |
| GOTERM_CC_FAT | GO:0044459~plasma membrane part | 161 | 2.4E-07 | 1.1E-05 |
| GOTERM_CC_FAT | GO:0005886~plasma membrane | 241 | 6.7E-06 | 2.6E-04 |
|  |  |  |  |  |
| Annotation Cluster 5 | Enrichment Score: 6.08 |  |  |  |
| Category | Term | Gene Count | P-Value | Benjamini-Hochberg P-value |
| UP_SEQ_FEATURE | topological domain:Cytoplasmic | 211 | 2.1E-10 | 1.2E-07 |
| UP_SEQ_FEATURE | topological domain:Extracellular | 178 | 3.0E-10 | 1.4E-07 |
| SP_PIR_KEYWORDS | membrane | 337 | 2.7E-09 | 2.2E-07 |
| SP_PIR_KEYWORDS | transmembrane | 270 | 1.9E-07 | 1.1E-05 |
| UP_SEQ_FEATURE | transmembrane region | 268 | 2.5E-07 | 6.6E-05 |
| GOTERM_CC_FAT | GO:0005886~plasma membrane | 241 | 6.7E-06 | 2.6E-04 |
| GOTERM_CC_FAT | GO:0031224~intrinsic to membrane | 297 | 4.7E-02 | 4.0E-01 |
| GOTERM_CC_FAT | GO:0016021~integral to membrane | 284 | 8.3E-02 | 5.5E-01 |
|  |  |  |  |  |
| Annotation Cluster 6 | Enrichment Score: 5.53 |  |  |  |
| Category | Term | Gene Count | P-Value | Benjamini-Hochberg P-value |
| SP_PIR_KEYWORDS | Immunoglobulin domain | 51 | 9.5E-10 | 8.9E-08 |
| UP_SEQ_FEATURE | domain:Ig-like C2-type 1 | 30 | 1.3E-09 | 5.3E-07 |
| UP_SEQ_FEATURE | domain:Ig-like C2-type 2 | 30 | 1.5E-09 | 5.1E-07 |
| INTERPRO | IPR013151:Immunoglobulin | 32 | 1.6E-09 | 1.9E-06 |
| UP_SEQ_FEATURE | domain:Ig-like C2-type 3 | 22 | 3.0E-08 | 8.8E-06 |
| INTERPRO | IPR007110:Immunoglobulin-like | 51 | 8.0E-08 | 4.8E-05 |
| INTERPRO | IPR003599:Immunoglobulin subtype | 39 | 8.2E-08 | 3.3E-05 |
| INTERPRO | IPR003598:Immunoglobulin subtype 2 | 28 | 4.5E-07 | 1.3E-04 |
| SMART | SM00409:IG | 39 | 6.8E-07 | 1.6E-04 |
| SMART | SM00408:IGc2 | 28 | 2.4E-06 | 2.8E-04 |
| INTERPRO | IPR013783:Immunoglobulin-like fold | 49 | 7.8E-06 | 1.9E-03 |
| INTERPRO | IPR013098:Immunoglobulin I-set | 19 | 4.1E-05 | 7.1E-03 |
| UP_SEQ_FEATURE | domain:Ig-like C2-type 5 | 10 | 4.1E-04 | 5.5E-02 |
| UP_SEQ_FEATURE | domain:Ig-like C2-type 6 | 8 | 7.2E-04 | 8.6E-02 |
| UP_SEQ_FEATURE | domain:Fibronectin type-III 4 | 9 | 4.0E-03 | 2.8E-01 |
| UP_SEQ_FEATURE | domain:Ig-like C2-type 4 | 10 | 4.2E-03 | 2.8E-01 |
| PIR_SUPERFAMILY | PIRSF002508:neural cell adhesion molecule-like protein | 3 | 1.6E-02 | 6.8E-01 |
| INTERPRO | IPR013106:Immunoglobulin V-set | 19 | 3.0E-02 | 6.2E-01 |
|  |  |  |  |  |
| Annotation Cluster 7 | Enrichment Score: 5.14 |  |  |  |
| Category | Term | Gene Count | P-Value | Benjamini-Hochberg P-value |
| GOTERM_MF_FAT | GO:0001871~pattern binding | 24 | 5.6E-07 | 4.9E-04 |
| GOTERM_MF_FAT | GO:0030247~polysaccharide binding | 24 | 5.6E-07 | 4.9E-04 |
| GOTERM_MF_FAT | GO:0030246~carbohydrate binding | 39 | 9.9E-07 | 4.3E-04 |
| GOTERM_MF_FAT | GO:0005539~glycosaminoglycan binding | 20 | 2.2E-05 | 4.8E-03 |
| GOTERM_MF_FAT | GO:0008201~heparin binding | 16 | 7.0E-05 | 1.0E-02 |
| SP_PIR_KEYWORDS | heparin-binding | 11 | 3.1E-04 | 9.2E-03 |
|  |  |  |  |  |
| Annotation Cluster 8 | Enrichment Score: 5.06 |  |  |  |
| Category | Term | Gene Count | P-Value | Benjamini-Hochberg P-value |
| GOTERM_BP_FAT | GO:0048514~blood vessel morphogenesis | 28 | 2.3E-06 | 8.4E-04 |
| GOTERM_BP_FAT | GO:0001944~vasculature development | 31 | 2.6E-06 | 7.8E-04 |
| GOTERM_BP_FAT | GO:0001568~blood vessel development | 30 | 4.7E-06 | 1.2E-03 |
| GOTERM_BP_FAT | GO:0001525~angiogenesis | 19 | 2.1E-04 | 1.7E-02 |
|  |  |  |  |  |
| Annotation Cluster 9 | Enrichment Score: 4.19 |  |  |  |
| Category | Term | Gene Count | P-Value | Benjamini-Hochberg P-value |
| GOTERM_BP_FAT | GO:0035295~tube development | 30 | 5.1E-07 | 3.8E-04 |
| GOTERM_BP_FAT | GO:0030324~lung development | 15 | 2.2E-04 | 1.6E-02 |
| GOTERM_BP_FAT | GO:0030323~respiratory tube development | 15 | 3.0E-04 | 1.9E-02 |
| GOTERM_BP_FAT | GO:0060541~respiratory system development | 15 | 5.4E-04 | 2.8E-02 |
|  |  |  |  |  |
| Annotation Cluster 10 | Enrichment Score: 3.97 |  |  |  |
| Category | Term | Gene Count | P-Value | Benjamini-Hochberg P-value |
| GOTERM_BP_FAT | GO:0040012~regulation of locomotion | 24 | 3.7E-05 | 4.6E-03 |
| GOTERM_BP_FAT | GO:0040017~positive regulation of locomotion | 16 | 5.2E-05 | 5.7E-03 |
| GOTERM_BP_FAT | GO:0051270~regulation of cell motion | 23 | 1.1E-04 | 1.1E-02 |
| GOTERM_BP_FAT | GO:0030334~regulation of cell migration | 21 | 1.4E-04 | 1.3E-02 |
| GOTERM_BP_FAT | GO:0051272~positive regulation of cell motion | 15 | 1.9E-04 | 1.6E-02 |
| GOTERM_BP_FAT | GO:0030335~positive regulation of cell migration | 14 | 2.6E-04 | 1.8E-02 |
|  |  |  |  |  |
| Annotation Cluster 11 | Enrichment Score: 3.84 |  |  |  |
| Category | Term | Gene Count | P-Value | Benjamini-Hochberg P-value |
| GOTERM_BP_FAT | GO:0048545~response to steroid hormone stimulus | 26 | 3.9E-06 | 1.1E-03 |
| GOTERM_BP_FAT | GO:0009719~response to endogenous stimulus | 41 | 7.8E-06 | 1.5E-03 |
| GOTERM_BP_FAT | GO:0009725~response to hormone stimulus | 38 | 1.1E-05 | 1.9E-03 |
| GOTERM_BP_FAT | GO:0010033~response to organic substance | 57 | 1.5E-04 | 1.3E-02 |
| GOTERM_BP_FAT | GO:0042493~response to drug | 24 | 2.2E-04 | 1.6E-02 |
| GOTERM_BP_FAT | GO:0043627~response to estrogen stimulus | 13 | 3.9E-03 | 1.1E-01 |
| GOTERM_BP_FAT | GO:0043434~response to peptide hormone stimulus | 14 | 3.0E-02 | 3.5E-01 |
|  |  |  |  |  |
| Annotation Cluster 12 | Enrichment Score: 3.78 |  |  |  |
| Category | Term | Gene Count | P-Value | Benjamini-Hochberg P-value |
| GOTERM_BP_FAT | GO:0006928~cell motion | 46 | 6.3E-06 | 1.3E-03 |
| GOTERM_BP_FAT | GO:0016477~cell migration | 28 | 2.7E-04 | 1.8E-02 |
| GOTERM_BP_FAT | GO:0051674~localization of cell | 29 | 6.6E-04 | 3.3E-02 |
| GOTERM_BP_FAT | GO:0048870~cell motility | 29 | 6.6E-04 | 3.3E-02 |
|  |  |  |  |  |
| Annotation Cluster 13 | Enrichment Score: 3.65 |  |  |  |
| Category | Term | Gene Count | P-Value | Benjamini-Hochberg P-value |
| GOTERM_BP_FAT | GO:0009611~response to wounding | 50 | 4.8E-06 | 1.1E-03 |
| GOTERM_BP_FAT | GO:0006952~defense response | 49 | 4.0E-04 | 2.2E-02 |
| GOTERM_BP_FAT | GO:0006954~inflammatory response | 27 | 6.0E-03 | 1.5E-01 |
|  |  |  |  |  |
| Annotation Cluster 14 | Enrichment Score: 3.63 |  |  |  |
| Category | Term | Gene Count | P-Value | Benjamini-Hochberg P-value |
| GOTERM_CC_FAT | GO:0000267~cell fraction | 86 | 1.9E-05 | 6.1E-04 |
| GOTERM_CC_FAT | GO:0005626~insoluble fraction | 64 | 8.0E-04 | 2.1E-02 |
| GOTERM_CC_FAT | GO:0005624~membrane fraction | 62 | 8.6E-04 | 2.1E-02 |
|  |  |  |  |  |
| Annotation Cluster 15 | Enrichment Score: 3.53 |  |  |  |
| Category | Term | Gene Count | P-Value | Benjamini-Hochberg P-value |
| GOTERM_BP_FAT | GO:0048545~response to steroid hormone stimulus | 26 | 3.9E-06 | 1.1E-03 |
| GOTERM_BP_FAT | GO:0051384~response to glucocorticoid stimulus | 13 | 2.7E-04 | 1.8E-02 |
| GOTERM_BP_FAT | GO:0031960~response to corticosteroid stimulus | 13 | 6.1E-04 | 3.1E-02 |
| GOTERM_BP_FAT | GO:0014070~response to organic cyclic substance | 13 | 1.2E-02 | 2.1E-01 |
|  |  |  |  |  |
| Annotation Cluster 16 | Enrichment Score: 3.44 |  |  |  |
| Category | Term | Gene Count | P-Value | Benjamini-Hochberg P-value |
| GOTERM_CC_FAT | GO:0044420~extracellular matrix part | 19 | 2.0E-05 | 6.0E-04 |
| GOTERM_CC_FAT | GO:0005604~basement membrane | 13 | 4.7E-04 | 1.3E-02 |
| SP_PIR_KEYWORDS | basement membrane | 7 | 5.0E-03 | 6.2E-02 |
|  |  |  |  |  |
| Annotation Cluster 17 | Enrichment Score: 3.29 |  |  |  |
| Category | Term | Gene Count | P-Value | Benjamini-Hochberg P-value |
| GOTERM_BP_FAT | GO:0042445~hormone metabolic process | 18 | 8.8E-06 | 1.6E-03 |
| GOTERM_BP_FAT | GO:0010817~regulation of hormone levels | 18 | 7.6E-04 | 3.6E-02 |
| GOTERM_BP_FAT | GO:0034754~cellular hormone metabolic process | 8 | 2.0E-02 | 2.8E-01 |
|  |  |  |  |  |
| Annotation Cluster 18 | Enrichment Score: 3.12 |  |  |  |
| Category | Term | Gene Count | P-Value | Benjamini-Hochberg P-value |
| UP_SEQ_FEATURE | domain:EGF-like 3 | 14 | 1.2E-05 | 2.5E-03 |
| SP_PIR_KEYWORDS | egf-like domain | 24 | 8.7E-05 | 3.2E-03 |
| INTERPRO | IPR006210:EGF-like | 23 | 9.4E-05 | 1.2E-02 |
| INTERPRO | IPR000742:EGF-like, type 3 | 22 | 1.6E-04 | 1.6E-02 |
| INTERPRO | IPR013032:EGF-like region, conserved site | 28 | 2.9E-04 | 2.5E-02 |
| SMART | SM00181:EGF | 23 | 3.3E-04 | 1.1E-02 |
| INTERPRO | IPR000152:EGF-type aspartate/asparagine hydroxylation conserved site | 14 | 3.6E-04 | 2.7E-02 |
| UP_SEQ_FEATURE | domain:EGF-like 1 | 15 | 4.7E-04 | 5.9E-02 |
| INTERPRO | IPR006209:EGF | 16 | 5.3E-04 | 3.7E-02 |
| UP_SEQ_FEATURE | domain:EGF-like 2 | 12 | 1.0E-03 | 1.2E-01 |
| INTERPRO | IPR018097:EGF-like calcium-binding, conserved site | 12 | 3.6E-03 | 1.9E-01 |
| INTERPRO | IPR001881:EGF-like calcium-binding | 12 | 3.6E-03 | 1.9E-01 |
| SMART | SM00179:EGF_CA | 12 | 7.1E-03 | 1.5E-01 |
| UP_SEQ_FEATURE | domain:EGF-like 2; calcium-binding | 7 | 3.1E-02 | 8.0E-01 |
| INTERPRO | IPR013091:EGF calcium-binding | 8 | 4.1E-02 | 6.9E-01 |
|  |  |  |  |  |
| Annotation Cluster 19 | Enrichment Score: 3.00 |  |  |  |
| Category | Term | Gene Count | P-Value | Benjamini-Hochberg P-value |
| GOTERM_BP_FAT | GO:0003013~circulatory system process | 26 | 2.2E-06 | 9.3E-04 |
| GOTERM_BP_FAT | GO:0008015~blood circulation | 26 | 2.2E-06 | 9.3E-04 |
| GOTERM_BP_FAT | GO:0003018~vascular process in circulatory system | 11 | 3.4E-04 | 2.0E-02 |
| GOTERM_BP_FAT | GO:0008217~regulation of blood pressure | 13 | 2.6E-03 | 8.9E-02 |
| GOTERM_BP_FAT | GO:0035150~regulation of tube size | 9 | 3.2E-03 | 1.0E-01 |
| GOTERM_BP_FAT | GO:0050880~regulation of blood vessel size | 9 | 3.2E-03 | 1.0E-01 |
| GOTERM_BP_FAT | GO:0042311~vasodilation | 4 | 1.2E-01 | 6.7E-01 |
| GOTERM_BP_FAT | GO:0042310~vasoconstriction | 3 | 1.9E-01 | 7.8E-01 |
|  |  |  |  |  |
| Annotation Cluster 20 | Enrichment Score: 2.87 |  |  |  |
| Category | Term | Gene Count | P-Value | Benjamini-Hochberg P-value |
| UP_SEQ_FEATURE | domain:Sema | 9 | 2.9E-05 | 5.7E-03 |
| INTERPRO | IPR001627:Semaphorin/CD100 antigen | 9 | 4.7E-05 | 7.1E-03 |
| SMART | SM00630:Sema | 9 | 9.1E-05 | 7.0E-03 |
| INTERPRO | IPR003659:Plexin/semaphorin/integrin | 10 | 1.4E-04 | 1.5E-02 |
| SMART | SM00423:PSI | 10 | 2.8E-04 | 1.6E-02 |
| INTERPRO | IPR002165:Plexin | 7 | 2.2E-03 | 1.3E-01 |
| PIR_SUPERFAMILY | PIRSF005526:semaphorin | 5 | 5.4E-03 | 6.8E-01 |
| KEGG_PATHWAY | hsa04360:Axon guidance | 15 | 7.5E-03 | 6.9E-01 |
| UP_SEQ_FEATURE | compositionally biased region:Arg/Lys-rich (basic) | 5 | 7.2E-02 | 9.5E-01 |
| INTERPRO | IPR015943:WD40/YVTN repeat-like | 13 | 6.9E-01 | 1.0E+00 |
|  |  |  |  |  |
| Annotation Cluster 21 | Enrichment Score: 2.69 |  |  |  |
| Category | Term | Gene Count | P-Value | Benjamini-Hochberg P-value |
| GOTERM_BP_FAT | GO:0006979~response to oxidative stress | 22 | 3.0E-05 | 3.8E-03 |
| GOTERM_BP_FAT | GO:0000302~response to reactive oxygen species | 10 | 8.5E-03 | 1.8E-01 |
| GOTERM_BP_FAT | GO:0010035~response to inorganic substance | 17 | 3.3E-02 | 3.7E-01 |
|  |  |  |  |  |
| Annotation Cluster 22 | Enrichment Score: 2.61 |  |  |  |
| Category | Term | Gene Count | P-Value | Benjamini-Hochberg P-value |
| INTERPRO | IPR008957:Fibronectin, type III-like fold | 23 | 2.4E-05 | 4.9E-03 |
| UP_SEQ_FEATURE | domain:Fibronectin type-III 2 | 17 | 1.0E-04 | 1.8E-02 |
| UP_SEQ_FEATURE | domain:Fibronectin type-III 1 | 17 | 1.1E-04 | 1.8E-02 |
| INTERPRO | IPR003961:Fibronectin, type III | 21 | 3.2E-04 | 2.6E-02 |
| SMART | SM00060:FN3 | 21 | 1.0E-03 | 2.9E-02 |
| UP_SEQ_FEATURE | domain:Fibronectin type-III 4 | 9 | 4.0E-03 | 2.8E-01 |
| UP_SEQ_FEATURE | domain:Fibronectin type-III 3 | 10 | 7.5E-03 | 4.3E-01 |
| UP_SEQ_FEATURE | domain:Fibronectin type-III 5 | 7 | 1.0E-02 | 5.3E-01 |
| UP_SEQ_FEATURE | domain:Fibronectin type-III 8 | 4 | 7.6E-02 | 9.5E-01 |
| UP_SEQ_FEATURE | domain:Fibronectin type-III 7 | 4 | 7.6E-02 | 9.5E-01 |
| UP_SEQ_FEATURE | domain:Fibronectin type-III 6 | 4 | 1.4E-01 | 9.9E-01 |
|  |  |  |  |  |
| Annotation Cluster 23 | Enrichment Score: 2.51 |  |  |  |
| Category | Term | Gene Count | P-Value | Benjamini-Hochberg P-value |
| GOTERM_BP_FAT | GO:0001655~urogenital system development | 18 | 1.5E-05 | 2.4E-03 |
| GOTERM_BP_FAT | GO:0001822~kidney development | 16 | 4.0E-05 | 4.8E-03 |
| GOTERM_BP_FAT | GO:0001657~ureteric bud development | 9 | 1.8E-04 | 1.5E-02 |
| GOTERM_BP_FAT | GO:0060429~epithelium development | 24 | 4.6E-04 | 2.4E-02 |
| GOTERM_BP_FAT | GO:0001656~metanephros development | 9 | 9.2E-04 | 4.1E-02 |
| GOTERM_BP_FAT | GO:0002009~morphogenesis of an epithelium | 13 | 2.8E-03 | 9.5E-02 |
| GOTERM_BP_FAT | GO:0035239~tube morphogenesis | 14 | 6.9E-03 | 1.6E-01 |
| GOTERM_BP_FAT | GO:0048754~branching morphogenesis of a tube | 9 | 1.1E-02 | 2.0E-01 |
| GOTERM_BP_FAT | GO:0048729~tissue morphogenesis | 17 | 1.1E-02 | 2.0E-01 |
| GOTERM_BP_FAT | GO:0060675~ureteric bud morphogenesis | 5 | 1.8E-02 | 2.7E-01 |
| GOTERM_BP_FAT | GO:0001658~branching involved in ureteric bud morphogenesis | 5 | 1.8E-02 | 2.7E-01 |
| GOTERM_BP_FAT | GO:0001763~morphogenesis of a branching structure | 9 | 2.3E-02 | 3.0E-01 |
| GOTERM_BP_FAT | GO:0060562~epithelial tube morphogenesis | 8 | 3.8E-02 | 3.9E-01 |
| GOTERM_BP_FAT | GO:0001569~patterning of blood vessels | 3 | 2.6E-01 | 8.6E-01 |
|  |  |  |  |  |
| Annotation Cluster 24 | Enrichment Score: 2.50 |  |  |  |
| Category | Term | Gene Count | P-Value | Benjamini-Hochberg P-value |
| GOTERM_BP_FAT | GO:0032570~response to progesterone stimulus | 8 | 2.3E-05 | 3.2E-03 |
| GOTERM_BP_FAT | GO:0034097~response to cytokine stimulus | 11 | 3.9E-03 | 1.1E-01 |
| BIOCARTA | h_il2rbPathway:IL-2 Receptor Beta Chain in T cell Activation | 4 | 3.7E-01 | 1.0E+00 |
