# Supplementary material for: Vitamin D Modulates Expression of the Airway Smooth Muscle Transcriptome in Fatal Asthma
Source: PLoS One. 2015 Jul 24;10(7):e0134057. doi: 10.1371/journal.pone.0134057 (PMC4514847; doi:10.1371/journal.pone.0134057)
Supplement: S2 Table — Categories selected were from clusters with enrichment scores >2.50 and with individual Benjamini-Hochberg corrected p-values <0.05 that correspond to known asthma-related structures and processes, plus two categories that met these criteria in the vitamin D treated cells vs. those at baseline. Genes listed were the differentially expressed ones for the corresponding category. (DOCX) [file pone.0134057.s007.docx]

| Annotation Category | Annotation Term | Number of Genes | Genes | Benjamini-Hochberg P-value |
| --- | --- | --- | --- | --- |
| GOTERM_CC_FAT | GO:0031012~extracellular matrix | 58 | *ACAN, ADAMTS12, ADAMTS14, ADAMTS5, ADAMTSL4, AGRN, APLP1, BMP4, CHI3L1, CHL1, CILP, COL14A1, COL4A1, COL4A5, COL4A6, COL8A2, CTHRC1, DAG1, DPT, EGFL6, ELN, EMILIN1, EMILIN2, ENTPD1, F3, FBN2, FGF1, FGF9, FLRT3, FMOD, GPC1, GPC3, GPC6, HAPLN1, LGALS3BP, LOX, LTBP1, MAMDC2, MFAP4, MFAP5, MMP11, MMP12, MMP23B, NAV2, NOV, NPNT, NTN4, POSTN, PRELP, RELN, SCUBE3, SPARCL1, SPON1, TFPI2, TGFB2, VIT, VWA1, WNT2* | 1.2E-13 |
| SP_PIR_KEYWORDS | Immunoglobulin domain | 51 | *ACAN, ALCAM, ALPK2, CADM1, CHL1, CILP, CNTN1, CRLF1, FGFR2, FGFR4, FLT1, FSTL5, GPR125, HAPLN1, HLA-A, IGFBP7, IGSF10, IL18R1, IL1R1, JAM2, KIRREL3, KIT, L1CAM, LRIG1, LRIG3, MALT1, MERTK, MMP23B, NFASC, NTM, PDGFRL, PSG1, PSG2, PSG4, PSG5, PSG9, PTPRD, PVR, ROR2, SCN2B, SCN4B, SDK1, SEMA3A, SEMA3C, SEMA3D, SEMA3E, SEMA7A, TEK, THY1, TREML3, VCAM1* | 8.9E-08 |
| GOTERM_BP_FAT | GO:0048545~response to steroid hormone stimulus | 26 | *A2M, ALDH2, BCL2, BMP4, CA9, CAV2, CCL2, DUSP1, ENO2, FOS, IGFBP7, JUNB, LDLR, LOX, MFGE8, NGF, OXTR, PTGS1, PTGS2, SDC1, SLC9A3, SOCS1, SOCS3, TGFB2, WFDC1, WNT2* | 1.1E-03 |
| GOTERM_BP_FAT | GO:0009611~response to wounding | 50 | *A2M, BCL2, BDKRB1, BLNK, C3, C7, CCL13, CCL2, CD302, CD36, CD55, CLU, DSP, ELK3, ENTPD1, F10, F3, FGF2, FOS, ID3, IGSF10, IL15, IL20RB, IL8, INA, LOX, MAP2K3, MDK, MECOM, MYH10, NFKBIZ, NGF, OLR1, PAPSS2, PDPN, PROS1, PTX3, RTN4RL2, SCARB1, SCG2, SCN9A, SDC1, SERPINA3, SERPINB2, SERPINE1, SYT7, TFPI2, TGFB2, TNFAIP6, TNFSF4* | 1.1E-03 |
| GOTERM_BP_FAT | GO:0006979~response to oxidative stress | 22 | *APOE, BCL2, CLU, CYGB, DUSP1, ERCC6, FOS, G6PD, GCLM, NAPRT1, NQO1, OLR1, PTGS1, PTGS2, PYCR1, SCARA3, SDC1, SERPINE1, SNCA, SRXN1, STAT1, UCP2* | 3.8E-03 |
| GOTERM_BP_FAT | GO:0030324~lung development | 15 | *BMP4, CAV2, DHCR7, FGF1, FGF2, FGF9, FGFR4, FOXA1, GATA6, LOX, MAN2A1, NFIB, PDPN, TBX4, WNT2* | 0.016 |
| GOTERM_MF_FAT | GO:0005125~cytokine activity | 18 | *BMP4, CCL13, CCL2, CXCL12, FAM3C, GDF10, GDF5, GDF6, GREM2, IL15, IL32, IL8, SCG2, TGFB2, TNFSF10, TNFSF13B, TNFSF15, TNFSF4* | 0.22 |
| GOTERM_MF_FAT | GO:0008009~chemokine activity | 4 | *CCL13, CCL2, CXCL12, IL8* | 0.94 |
