# Supplementary material for: Vitamin D Modulates Expression of the Airway Smooth Muscle Transcriptome in Fatal Asthma
Source: PLoS One. 2015 Jul 24;10(7):e0134057. doi: 10.1371/journal.pone.0134057 (PMC4514847; doi:10.1371/journal.pone.0134057)
Supplement: S3 Table — Clusters with enrichment scores >1.5 are shown. Individual P-values listed correspond to EASE Scores, or modified Fisher Exact P-Values computed by DAVID. (DOCX) [file pone.0134057.s008.docx]

| Annotation Cluster 1 | Enrichment Score: 13.29 |  |  |  |
| --- | --- | --- | --- | --- |
| Category | Term | Gene Count | P-Value | Benjamini-Hochberg P-value |
| SP_PIR_KEYWORDS | signal | 234 | 1.1E-17 | 5.9E-15 |
| SP_PIR_KEYWORDS | glycoprotein | 288 | 1.3E-17 | 3.4E-15 |
| UP_SEQ_FEATURE | signal peptide | 234 | 2.4E-17 | 5.8E-14 |
| SP_PIR_KEYWORDS | Secreted | 141 | 6.0E-15 | 1.0E-12 |
| GOTERM_CC_FAT | GO:0044421~extracellular region part | 103 | 2.2E-14 | 8.4E-12 |
| UP_SEQ_FEATURE | glycosylation site:N-linked (GlcNAc...) | 269 | 2.8E-14 | 3.4E-11 |
| GOTERM_CC_FAT | GO:0005576~extracellular region | 171 | 3.1E-14 | 6.0E-12 |
| SP_PIR_KEYWORDS | disulfide bond | 193 | 1.4E-10 | 1.8E-08 |
| UP_SEQ_FEATURE | disulfide bond | 185 | 1.1E-09 | 6.4E-07 |
| GOTERM_CC_FAT | GO:0005615~extracellular space | 71 | 2.3E-09 | 3.0E-07 |
|  |  |  |  |  |
| Annotation Cluster 2 | Enrichment Score: 7.43 |  |  |  |
| Category | Term | Gene Count | P-Value | Benjamini-Hochberg P-value |
| GOTERM_BP_FAT | GO:0009725~response to hormone stimulus | 48 | 6.1E-10 | 9.4E-07 |
| GOTERM_BP_FAT | GO:0009719~response to endogenous stimulus | 49 | 5.3E-09 | 5.5E-06 |
| GOTERM_BP_FAT | GO:0010033~response to organic substance | 68 | 1.1E-07 | 3.5E-05 |
| GOTERM_BP_FAT | GO:0048545~response to steroid hormone stimulus | 26 | 5.0E-06 | 9.7E-04 |
|  |  |  |  |  |
| Annotation Cluster 3 | Enrichment Score: 6.17 |  |  |  |
| Category | Term | Gene Count | P-Value | Benjamini-Hochberg P-value |
| GOTERM_BP_FAT | GO:0009611~response to wounding | 63 | 4.8E-11 | 1.5E-07 |
| GOTERM_BP_FAT | GO:0006954~inflammatory response | 37 | 2.3E-06 | 5.1E-04 |
| GOTERM_BP_FAT | GO:0006952~defense response | 46 | 2.9E-03 | 6.5E-02 |
|  |  |  |  |  |
| Annotation Cluster 4 | Enrichment Score: 5.49 |  |  |  |
| Category | Term | Gene Count | P-Value | Benjamini-Hochberg P-value |
| GOTERM_BP_FAT | GO:0043069~negative regulation of programmed cell death | 45 | 8.5E-09 | 6.6E-06 |
| GOTERM_BP_FAT | GO:0060548~negative regulation of cell death | 45 | 9.3E-09 | 5.8E-06 |
| GOTERM_BP_FAT | GO:0043066~negative regulation of apoptosis | 44 | 1.7E-08 | 8.6E-06 |
| GOTERM_BP_FAT | GO:0043067~regulation of programmed cell death | 76 | 2.3E-08 | 1.0E-05 |
| GOTERM_BP_FAT | GO:0010941~regulation of cell death | 76 | 2.7E-08 | 1.0E-05 |
| GOTERM_BP_FAT | GO:0042981~regulation of apoptosis | 75 | 3.5E-08 | 1.2E-05 |
| GOTERM_BP_FAT | GO:0006916~anti-apoptosis | 28 | 1.8E-06 | 4.8E-04 |
| GOTERM_BP_FAT | GO:0043065~positive regulation of apoptosis | 37 | 7.8E-04 | 2.8E-02 |
| GOTERM_BP_FAT | GO:0043068~positive regulation of programmed cell death | 37 | 8.8E-04 | 3.1E-02 |
| GOTERM_BP_FAT | GO:0010942~positive regulation of cell death | 37 | 9.6E-04 | 3.2E-02 |
| GOTERM_BP_FAT | GO:0006917~induction of apoptosis | 27 | 5.9E-03 | 1.0E-01 |
| GOTERM_BP_FAT | GO:0012502~induction of programmed cell death | 27 | 6.1E-03 | 1.1E-01 |
|  |  |  |  |  |
| Annotation Cluster 5 | Enrichment Score: 5.07 |  |  |  |
| Category | Term | Gene Count | P-Value | Benjamini-Hochberg P-value |
| GOTERM_CC_FAT | GO:0031012~extracellular matrix | 43 | 3.8E-08 | 3.7E-06 |
| GOTERM_CC_FAT | GO:0005578~proteinaceous extracellular matrix | 36 | 6.3E-06 | 3.5E-04 |
| SP_PIR_KEYWORDS | extracellular matrix | 27 | 1.3E-05 | 1.0E-03 |
| GOTERM_CC_FAT | GO:0044420~extracellular matrix part | 15 | 1.6E-03 | 3.9E-02 |
|  |  |  |  |  |
| Annotation Cluster 6 | Enrichment Score: 4.99 |  |  |  |
| Category | Term | Gene Count | P-Value | Benjamini-Hochberg P-value |
| GOTERM_BP_FAT | GO:0009991~response to extracellular stimulus | 29 | 2.2E-06 | 5.1E-04 |
| GOTERM_BP_FAT | GO:0031667~response to nutrient levels | 26 | 7.9E-06 | 1.4E-03 |
| GOTERM_BP_FAT | GO:0007584~response to nutrient | 21 | 1.1E-05 | 1.7E-03 |
| GOTERM_BP_FAT | GO:0033273~response to vitamin | 13 | 5.9E-05 | 4.7E-03 |
|  |  |  |  |  |
| Annotation Cluster 7 | Enrichment Score: 4.89 |  |  |  |
| Category | Term | Gene Count | P-Value | Benjamini-Hochberg P-value |
| GOTERM_CC_FAT | GO:0044459~plasma membrane part | 159 | 1.0E-07 | 7.9E-06 |
| GOTERM_CC_FAT | GO:0031226~intrinsic to plasma membrane | 94 | 5.9E-06 | 3.8E-04 |
| GOTERM_CC_FAT | GO:0005887~integral to plasma membrane | 89 | 3.8E-05 | 1.5E-03 |
| GOTERM_CC_FAT | GO:0005886~plasma membrane | 220 | 1.2E-03 | 3.0E-02 |
|  |  |  |  |  |
| Annotation Cluster 8 | Enrichment Score: 4.16 |  |  |  |
| Category | Term | Gene Count | P-Value | Benjamini-Hochberg P-value |
| GOTERM_BP_FAT | GO:0048514~blood vessel morphogenesis | 26 | 2.6E-05 | 3.2E-03 |
| GOTERM_BP_FAT | GO:0001944~vasculature development | 29 | 2.7E-05 | 3.2E-03 |
| GOTERM_BP_FAT | GO:0001568~blood vessel development | 28 | 4.6E-05 | 3.9E-03 |
| GOTERM_BP_FAT | GO:0001525~angiogenesis | 18 | 7.1E-04 | 2.6E-02 |
|  |  |  |  |  |
| Annotation Cluster 9 | Enrichment Score: 4.10 |  |  |  |
| Category | Term | Gene Count | P-Value | Benjamini-Hochberg P-value |
| GOTERM_BP_FAT | GO:0008219~cell death | 60 | 3.2E-05 | 3.5E-03 |
| GOTERM_BP_FAT | GO:0016265~death | 60 | 3.8E-05 | 3.5E-03 |
| GOTERM_BP_FAT | GO:0006915~apoptosis | 51 | 9.6E-05 | 6.6E-03 |
| GOTERM_BP_FAT | GO:0012501~programmed cell death | 51 | 1.4E-04 | 8.6E-03 |
| SP_PIR_KEYWORDS | Apoptosis | 33 | 2.1E-04 | 8.3E-03 |
|  |  |  |  |  |
| Annotation Cluster 10 | Enrichment Score: 4.03 |  |  |  |
| Category | Term | Gene Count | P-Value | Benjamini-Hochberg P-value |
| GOTERM_BP_FAT | GO:0042060~wound healing | 27 | 1.4E-06 | 3.9E-04 |
| GOTERM_BP_FAT | GO:0007599~hemostasis | 17 | 5.2E-05 | 4.3E-03 |
| GOTERM_BP_FAT | GO:0007596~blood coagulation | 16 | 9.7E-05 | 6.5E-03 |
| GOTERM_BP_FAT | GO:0050817~coagulation | 16 | 9.7E-05 | 6.5E-03 |
| GOTERM_BP_FAT | GO:0050878~regulation of body fluid levels | 18 | 4.0E-04 | 1.6E-02 |
| SP_PIR_KEYWORDS | blood coagulation | 8 | 2.4E-03 | 3.9E-02 |
|  |  |  |  |  |
| Annotation Cluster 11 | Enrichment Score: 3.86 |  |  |  |
| Category | Term | Gene Count | P-Value | Benjamini-Hochberg P-value |
| GOTERM_MF_FAT | GO:0005539~glycosaminoglycan binding | 20 | 2.0E-05 | 4.1E-03 |
| GOTERM_MF_FAT | GO:0001871~pattern binding | 20 | 7.6E-05 | 1.2E-02 |
| GOTERM_MF_FAT | GO:0030247~polysaccharide binding | 20 | 7.6E-05 | 1.2E-02 |
| GOTERM_MF_FAT | GO:0030246~carbohydrate binding | 33 | 1.8E-04 | 1.9E-02 |
| SP_PIR_KEYWORDS | heparin-binding | 11 | 3.9E-04 | 1.0E-02 |
| GOTERM_MF_FAT | GO:0008201~heparin binding | 14 | 8.1E-04 | 6.4E-02 |
|  |  |  |  |  |
| Annotation Cluster 12 | Enrichment Score: 3.85 |  |  |  |
| Category | Term | Gene Count | P-Value | Benjamini-Hochberg P-value |
| GOTERM_BP_FAT | GO:0006928~cell motion | 46 | 9.1E-06 | 1.6E-03 |
| GOTERM_BP_FAT | GO:0016477~cell migration | 29 | 1.4E-04 | 8.7E-03 |
| GOTERM_BP_FAT | GO:0050900~leukocyte migration | 11 | 3.3E-04 | 1.5E-02 |
| GOTERM_BP_FAT | GO:0048870~cell motility | 30 | 3.7E-04 | 1.6E-02 |
| GOTERM_BP_FAT | GO:0051674~localization of cell | 30 | 3.7E-04 | 1.6E-02 |
|  |  |  |  |  |
| Annotation Cluster 13 | Enrichment Score: 3.76 |  |  |  |
| Category | Term | Gene Count | P-Value | Benjamini-Hochberg P-value |
| SP_PIR_KEYWORDS | lipoprotein | 50 | 5.8E-05 | 3.4E-03 |
| UP_SEQ_FEATURE | lipid moiety-binding region:S-palmitoyl cysteine | 20 | 2.9E-04 | 1.3E-01 |
| SP_PIR_KEYWORDS | palmitate | 22 | 3.1E-04 | 9.0E-03 |
|  |  |  |  |  |
| Annotation Cluster 14 | Enrichment Score: 3.69 |  |  |  |
| Category | Term | Gene Count | P-Value | Benjamini-Hochberg P-value |
| GOTERM_CC_FAT | GO:0005624~membrane fraction | 64 | 1.4E-04 | 4.8E-03 |
| GOTERM_CC_FAT | GO:0005626~insoluble fraction | 65 | 2.2E-04 | 7.0E-03 |
| GOTERM_CC_FAT | GO:0000267~cell fraction | 79 | 2.8E-04 | 8.3E-03 |
|  |  |  |  |  |
| Annotation Cluster 15 | Enrichment Score: 3.49 |  |  |  |
| Category | Term | Gene Count | P-Value | Benjamini-Hochberg P-value |
| SP_PIR_KEYWORDS | cell adhesion | 35 | 2.9E-04 | 8.9E-03 |
| GOTERM_BP_FAT | GO:0007155~cell adhesion | 55 | 3.3E-04 | 1.5E-02 |
| GOTERM_BP_FAT | GO:0022610~biological adhesion | 55 | 3.4E-04 | 1.5E-02 |
|  |  |  |  |  |
| Annotation Cluster 16 | Enrichment Score: 3.47 |  |  |  |
| Category | Term | Gene Count | P-Value | Benjamini-Hochberg P-value |
| GOTERM_BP_FAT | GO:0048878~chemical homeostasis | 48 | 1.3E-05 | 1.9E-03 |
| GOTERM_BP_FAT | GO:0006875~cellular metal ion homeostasis | 25 | 2.2E-05 | 2.9E-03 |
| GOTERM_BP_FAT | GO:0055066~di-, tri-valent inorganic cation homeostasis | 28 | 3.0E-05 | 3.4E-03 |
| GOTERM_BP_FAT | GO:0030005~cellular di-, tri-valent inorganic cation homeostasis | 27 | 3.3E-05 | 3.5E-03 |
| GOTERM_BP_FAT | GO:0030003~cellular cation homeostasis | 29 | 3.3E-05 | 3.3E-03 |
| GOTERM_BP_FAT | GO:0055065~metal ion homeostasis | 25 | 4.6E-05 | 3.9E-03 |
| GOTERM_BP_FAT | GO:0006874~cellular calcium ion homeostasis | 23 | 6.3E-05 | 4.9E-03 |
| GOTERM_BP_FAT | GO:0055074~calcium ion homeostasis | 23 | 9.5E-05 | 6.7E-03 |
| GOTERM_BP_FAT | GO:0055080~cation homeostasis | 30 | 1.1E-04 | 7.1E-03 |
| GOTERM_BP_FAT | GO:0042592~homeostatic process | 57 | 6.0E-04 | 2.3E-02 |
| GOTERM_BP_FAT | GO:0006873~cellular ion homeostasis | 33 | 1.1E-03 | 3.4E-02 |
| GOTERM_BP_FAT | GO:0055082~cellular chemical homeostasis | 33 | 1.4E-03 | 4.2E-02 |
| GOTERM_BP_FAT | GO:0050801~ion homeostasis | 34 | 2.3E-03 | 5.6E-02 |
| GOTERM_BP_FAT | GO:0019725~cellular homeostasis | 37 | 3.1E-03 | 6.7E-02 |
| GOTERM_BP_FAT | GO:0007204~elevation of cytosolic calcium ion concentration | 13 | 6.3E-03 | 1.1E-01 |
| GOTERM_BP_FAT | GO:0051480~cytosolic calcium ion homeostasis | 13 | 1.1E-02 | 1.5E-01 |
| KEGG_PATHWAY | hsa04020:Calcium signaling pathway | 16 | 8.7E-02 | 4.3E-01 |
|  |  |  |  |  |
| Annotation Cluster 17 | Enrichment Score: 3.44 |  |  |  |
| Category | Term | Gene Count | P-Value | Benjamini-Hochberg P-value |
| GOTERM_BP_FAT | GO:0010035~response to inorganic substance | 25 | 4.6E-05 | 3.9E-03 |
| GOTERM_BP_FAT | GO:0006979~response to oxidative stress | 21 | 1.1E-04 | 7.2E-03 |
| GOTERM_BP_FAT | GO:0000302~response to reactive oxygen species | 13 | 2.1E-04 | 1.2E-02 |
| GOTERM_BP_FAT | GO:0042542~response to hydrogen peroxide | 8 | 1.7E-02 | 2.0E-01 |
|  |  |  |  |  |
| Annotation Cluster 18 | Enrichment Score: 3.41 |  |  |  |
| Category | Term | Gene Count | P-Value | Benjamini-Hochberg P-value |
| INTERPRO | IPR001811:Small chemokine, interleukin-8-like | 12 | 1.3E-06 | 1.5E-03 |
| SMART | SM00199:SCY | 12 | 4.2E-06 | 1.1E-03 |
| GOTERM_MF_FAT | GO:0008009~chemokine activity | 12 | 5.1E-06 | 2.1E-03 |
| GOTERM_MF_FAT | GO:0042379~chemokine receptor binding | 12 | 9.8E-06 | 2.7E-03 |
| PIR_SUPERFAMILY | PIRSF500572:small inducible cytokine, A2 type | 5 | 2.3E-05 | 8.5E-03 |
| GOTERM_MF_FAT | GO:0005125~cytokine activity | 23 | 8.3E-05 | 1.1E-02 |
| KEGG_PATHWAY | hsa04060:Cytokine-cytokine receptor interaction | 32 | 9.9E-05 | 1.6E-02 |
| INTERPRO | IPR001089:Small chemokine, C-X-C | 6 | 1.7E-04 | 5.0E-02 |
| KEGG_PATHWAY | hsa04621:NOD-like receptor signaling pathway | 13 | 1.9E-04 | 1.5E-02 |
| GOTERM_BP_FAT | GO:0042330~taxis | 20 | 2.3E-04 | 1.2E-02 |
| GOTERM_BP_FAT | GO:0006935~chemotaxis | 20 | 2.3E-04 | 1.2E-02 |
| INTERPRO | IPR002473:Small chemokine, C-X-C/Interleukin 8 | 6 | 2.6E-04 | 6.0E-02 |
| SP_PIR_KEYWORDS | cytokine | 20 | 2.7E-04 | 8.7E-03 |
| GOTERM_BP_FAT | GO:0007610~behavior | 41 | 2.8E-04 | 1.4E-02 |
| SP_PIR_KEYWORDS | inflammatory response | 12 | 4.0E-04 | 9.8E-03 |
| GOTERM_BP_FAT | GO:0007626~locomotory behavior | 27 | 6.8E-04 | 2.5E-02 |
| INTERPRO | IPR018048:Small chemokine, C-X-C, conserved site | 6 | 7.2E-04 | 9.0E-02 |
| PIR_SUPERFAMILY | PIRSF002522:CXC chemokine | 5 | 3.3E-03 | 4.6E-01 |
| SP_PIR_KEYWORDS | chemotaxis | 10 | 3.4E-03 | 4.9E-02 |
| BBID | 109.Chemokine_families | 8 | 8.5E-03 | 4.0E-01 |
| KEGG_PATHWAY | hsa04062:Chemokine signaling pathway | 20 | 1.2E-02 | 1.7E-01 |
| PIR_SUPERFAMILY | PIRSF500568:growth regulated protein, alpha/beta/gamma types | 3 | 1.2E-02 | 6.9E-01 |
| PIR_SUPERFAMILY | PIRSF001950:small inducible chemokine, C/CC types | 5 | 2.1E-02 | 6.7E-01 |
| SP_PIR_KEYWORDS | Pyrrolidone carboxylic acid | 8 | 2.3E-02 | 1.8E-01 |
| INTERPRO | IPR000827:Small chemokine, C-C group, conserved site | 5 | 2.4E-02 | 6.3E-01 |
|  |  |  |  |  |
| Annotation Cluster 19 | Enrichment Score: 3.23 |  |  |  |
| Category | Term | Gene Count | P-Value | Benjamini-Hochberg P-value |
| GOTERM_BP_FAT | GO:0001501~skeletal system development | 35 | 1.0E-05 | 1.7E-03 |
| GOTERM_BP_FAT | GO:0001503~ossification | 14 | 3.3E-03 | 7.0E-02 |
| GOTERM_BP_FAT | GO:0060348~bone development | 14 | 5.9E-03 | 1.0E-01 |
|  |  |  |  |  |
| Annotation Cluster 20 | Enrichment Score: 2.70 |  |  |  |
| Category | Term | Gene Count | P-Value | Benjamini-Hochberg P-value |
| GOTERM_BP_FAT | GO:0042330~taxis | 20 | 2.3E-04 | 1.2E-02 |
| GOTERM_BP_FAT | GO:0006935~chemotaxis | 20 | 2.3E-04 | 1.2E-02 |
| GOTERM_BP_FAT | GO:0050900~leukocyte migration | 11 | 3.3E-04 | 1.5E-02 |
| GOTERM_BP_FAT | GO:0030595~leukocyte chemotaxis | 8 | 1.6E-03 | 4.6E-02 |
| GOTERM_BP_FAT | GO:0060326~cell chemotaxis | 8 | 2.2E-03 | 5.6E-02 |
| GOTERM_BP_FAT | GO:0030593~neutrophil chemotaxis | 5 | 9.3E-03 | 1.4E-01 |
| BBID | 18.Cytokine_astocytes | 4 | 2.2E-01 | 1.0E+00 |
|  |  |  |  |  |
| Annotation Cluster 21 | Enrichment Score: 2.69 |  |  |  |
| Category | Term | Gene Count | P-Value | Benjamini-Hochberg P-value |
| SP_PIR_KEYWORDS | oxidoreductase | 42 | 5.7E-04 | 1.3E-02 |
| SP_PIR_KEYWORDS | nadp | 17 | 1.0E-03 | 2.0E-02 |
| GOTERM_BP_FAT | GO:0055114~oxidation reduction | 44 | 1.5E-02 | 1.8E-01 |
|  |  |  |  |  |
| Annotation Cluster 22 | Enrichment Score: 2.62 |  |  |  |
| Category | Term | Gene Count | P-Value | Benjamini-Hochberg P-value |
| GOTERM_BP_FAT | GO:0060541~respiratory system development | 14 | 1.9E-03 | 5.1E-02 |
| GOTERM_BP_FAT | GO:0035295~tube development | 22 | 2.0E-03 | 5.3E-02 |
| GOTERM_BP_FAT | GO:0030324~lung development | 13 | 2.6E-03 | 6.1E-02 |
| GOTERM_BP_FAT | GO:0030323~respiratory tube development | 13 | 3.4E-03 | 7.1E-02 |
|  |  |  |  |  |
| Annotation Cluster 23 | Enrichment Score: 2.59 |  |  |  |
| Category | Term | Gene Count | P-Value | Benjamini-Hochberg P-value |
| GOTERM_BP_FAT | GO:0002237~response to molecule of bacterial origin | 14 | 2.1E-04 | 1.2E-02 |
| GOTERM_BP_FAT | GO:0032496~response to lipopolysaccharide | 12 | 1.0E-03 | 3.4E-02 |
| GOTERM_BP_FAT | GO:0009617~response to bacterium | 15 | 7.9E-02 | 5.3E-01 |
|  |  |  |  |  |
| Annotation Cluster 24 | Enrichment Score: 2.58 |  |  |  |
| Category | Term | Gene Count | P-Value | Benjamini-Hochberg P-value |
| GOTERM_BP_FAT | GO:0048666~neuron development | 33 | 1.9E-04 | 1.1E-02 |
| GOTERM_BP_FAT | GO:0030182~neuron differentiation | 39 | 2.8E-04 | 1.4E-02 |
| GOTERM_BP_FAT | GO:0000902~cell morphogenesis | 33 | 4.5E-04 | 1.8E-02 |
| GOTERM_BP_FAT | GO:0000904~cell morphogenesis involved in differentiation | 25 | 6.5E-04 | 2.4E-02 |
| GOTERM_BP_FAT | GO:0031175~neuron projection development | 25 | 1.3E-03 | 4.0E-02 |
| GOTERM_BP_FAT | GO:0048812~neuron projection morphogenesis | 22 | 1.4E-03 | 4.1E-02 |
| GOTERM_BP_FAT | GO:0048667~cell morphogenesis involved in neuron differentiation | 21 | 2.5E-03 | 5.8E-02 |
| GOTERM_BP_FAT | GO:0032989~cellular component morphogenesis | 33 | 2.7E-03 | 6.2E-02 |
| GOTERM_BP_FAT | GO:0007409~axonogenesis | 19 | 5.2E-03 | 9.6E-02 |
| GOTERM_BP_FAT | GO:0048858~cell projection morphogenesis | 22 | 7.1E-03 | 1.2E-01 |
| GOTERM_BP_FAT | GO:0032990~cell part morphogenesis | 22 | 1.1E-02 | 1.5E-01 |
| GOTERM_BP_FAT | GO:0030030~cell projection organization | 27 | 3.0E-02 | 3.0E-01 |
| GOTERM_BP_FAT | GO:0007411~axon guidance | 9 | 1.4E-01 | 6.9E-01 |
