# Supplementary material for: Vitamin D Modulates Expression of the Airway Smooth Muscle Transcriptome in Fatal Asthma
Source: PLoS One. 2015 Jul 24;10(7):e0134057. doi: 10.1371/journal.pone.0134057 (PMC4514847; doi:10.1371/journal.pone.0134057)
Supplement: S4 Table — Clusters with enrichment scores >1.5 are shown. Individual P-values listed correspond to EASE Scores, or modified Fisher Exact P-Values computed by DAVID. (DOCX) [file pone.0134057.s009.docx]

| Annotation Cluster 1 | Enrichment Score: 18.00 |  |  |  |
| --- | --- | --- | --- | --- |
| Category | Term | Gene Count | P-Value | Benjamini-Hochberg P-value |
| SP_PIR_KEYWORDS | glycoprotein | 271 | 1.6E-24 | 7.5E-22 |
| SP_PIR_KEYWORDS | signal | 219 | 9.0E-23 | 2.2E-20 |
| UP_SEQ_FEATURE | signal peptide | 219 | 2.0E-22 | 4.2E-19 |
| UP_SEQ_FEATURE | glycosylation site:N-linked (GlcNAc...) | 254 | 5.4E-21 | 5.7E-18 |
| SP_PIR_KEYWORDS | Secreted | 136 | 8.0E-20 | 1.3E-17 |
| GOTERM_CC_FAT | GO:0005576~extracellular region | 161 | 3.0E-17 | 1.1E-14 |
| SP_PIR_KEYWORDS | disulfide bond | 172 | 9.5E-12 | 1.1E-09 |
| UP_SEQ_FEATURE | disulfide bond | 163 | 2.7E-10 | 1.9E-07 |
|  |  |  |  |  |
| Annotation Cluster 2 | Enrichment Score: 14.09 |  |  |  |
| Category | Term | Gene Count | P-Value | Benjamini-Hochberg P-value |
| GOTERM_CC_FAT | GO:0005576~extracellular region | 161 | 3.0E-17 | 1.1E-14 |
| GOTERM_CC_FAT | GO:0044421~extracellular region part | 98 | 2.4E-16 | 4.0E-14 |
| GOTERM_CC_FAT | GO:0005615~extracellular space | 68 | 7.2E-11 | 8.6E-09 |
|  |  |  |  |  |
| Annotation Cluster 3 | Enrichment Score: 7.05 |  |  |  |
| Category | Term | Gene Count | P-Value | Benjamini-Hochberg P-value |
| GOTERM_CC_FAT | GO:0031012~extracellular matrix | 43 | 5.3E-10 | 4.7E-08 |
| GOTERM_CC_FAT | GO:0005578~proteinaceous extracellular matrix | 38 | 2.4E-08 | 1.7E-06 |
| SP_PIR_KEYWORDS | extracellular matrix | 29 | 4.2E-08 | 4.0E-06 |
| GOTERM_CC_FAT | GO:0044420~extracellular matrix part | 16 | 1.2E-04 | 4.6E-03 |
|  |  |  |  |  |
| Annotation Cluster 4 | Enrichment Score: 6.74 |  |  |  |
| Category | Term | Gene Count | P-Value | Benjamini-Hochberg P-value |
| GOTERM_BP_FAT | GO:0007155~cell adhesion | 59 | 6.5E-08 | 1.9E-04 |
| GOTERM_BP_FAT | GO:0022610~biological adhesion | 59 | 6.8E-08 | 6.4E-05 |
| SP_PIR_KEYWORDS | cell adhesion | 37 | 1.3E-06 | 1.1E-04 |
|  |  |  |  |  |
| Annotation Cluster 5 | Enrichment Score: 5.20 |  |  |  |
| Category | Term | Gene Count | P-Value | Benjamini-Hochberg P-value |
| GOTERM_BP_FAT | GO:0009611~response to wounding | 49 | 6.7E-08 | 9.6E-05 |
| GOTERM_BP_FAT | GO:0006954~inflammatory response | 30 | 3.8E-05 | 3.6E-03 |
| GOTERM_BP_FAT | GO:0006952~defense response | 45 | 1.0E-04 | 6.6E-03 |
|  |  |  |  |  |
| Annotation Cluster 6 | Enrichment Score: 4.84 |  |  |  |
| Category | Term | Gene Count | P-Value | Benjamini-Hochberg P-value |
| GOTERM_BP_FAT | GO:0051272~positive regulation of cell motion | 16 | 6.6E-06 | 9.9E-04 |
| GOTERM_BP_FAT | GO:0040017~positive regulation of locomotion | 16 | 6.6E-06 | 9.9E-04 |
| GOTERM_BP_FAT | GO:0051270~regulation of cell motion | 23 | 8.0E-06 | 1.0E-03 |
| GOTERM_BP_FAT | GO:0030335~positive regulation of cell migration | 15 | 9.6E-06 | 1.2E-03 |
| GOTERM_BP_FAT | GO:0030334~regulation of cell migration | 20 | 4.0E-05 | 3.7E-03 |
| GOTERM_BP_FAT | GO:0040012~regulation of locomotion | 21 | 7.5E-05 | 6.1E-03 |
|  |  |  |  |  |
| Annotation Cluster 7 | Enrichment Score: 4.67 |  |  |  |
| Category | Term | Gene Count | P-Value | Benjamini-Hochberg P-value |
| GOTERM_BP_FAT | GO:0033273~response to vitamin | 15 | 2.2E-07 | 1.6E-04 |
| GOTERM_BP_FAT | GO:0009719~response to endogenous stimulus | 40 | 2.5E-07 | 1.5E-04 |
| GOTERM_BP_FAT | GO:0048545~response to steroid hormone stimulus | 25 | 5.9E-07 | 2.8E-04 |
| GOTERM_BP_FAT | GO:0009725~response to hormone stimulus | 36 | 1.3E-06 | 4.8E-04 |
| GOTERM_BP_FAT | GO:0010033~response to organic substance | 56 | 2.0E-06 | 6.3E-04 |
| GOTERM_BP_FAT | GO:0009991~response to extracellular stimulus | 26 | 2.0E-06 | 5.8E-04 |
| GOTERM_BP_FAT | GO:0007584~response to nutrient | 20 | 2.6E-06 | 6.7E-04 |
| GOTERM_BP_FAT | GO:0031667~response to nutrient levels | 24 | 3.3E-06 | 6.7E-04 |
| GOTERM_BP_FAT | GO:0043627~response to estrogen stimulus | 14 | 2.5E-04 | 1.3E-02 |
| GOTERM_BP_FAT | GO:0031960~response to corticosteroid stimulus | 12 | 5.0E-04 | 2.3E-02 |
| GOTERM_BP_FAT | GO:0051384~response to glucocorticoid stimulus | 11 | 9.6E-04 | 3.6E-02 |
| GOTERM_BP_FAT | GO:0033189~response to vitamin A | 8 | 1.2E-03 | 4.1E-02 |
| GOTERM_BP_FAT | GO:0042493~response to drug | 19 | 2.3E-03 | 6.7E-02 |
| GOTERM_BP_FAT | GO:0032526~response to retinoic acid | 6 | 9.0E-03 | 1.6E-01 |
|  |  |  |  |  |
| Annotation Cluster 8 | Enrichment Score: 4.17 |  |  |  |
| Category | Term | Gene Count | P-Value | Benjamini-Hochberg P-value |
| GOTERM_CC_FAT | GO:0044459~plasma membrane part | 136 | 1.8E-06 | 1.1E-04 |
| GOTERM_CC_FAT | GO:0031226~intrinsic to plasma membrane | 77 | 2.8E-04 | 8.3E-03 |
| GOTERM_CC_FAT | GO:0005887~integral to plasma membrane | 74 | 6.2E-04 | 1.6E-02 |
|  |  |  |  |  |
| Annotation Cluster 9 | Enrichment Score: 4.05 |  |  |  |
| Category | Term | Gene Count | P-Value | Benjamini-Hochberg P-value |
| GOTERM_BP_FAT | GO:0006928~cell motion | 40 | 1.3E-05 | 1.5E-03 |
| GOTERM_BP_FAT | GO:0016477~cell migration | 26 | 1.0E-04 | 6.5E-03 |
| GOTERM_BP_FAT | GO:0051674~localization of cell | 27 | 2.2E-04 | 1.2E-02 |
| GOTERM_BP_FAT | GO:0048870~cell motility | 27 | 2.2E-04 | 1.2E-02 |
|  |  |  |  |  |
| Annotation Cluster 10 | Enrichment Score: 3.84 |  |  |  |
| Category | Term | Gene Count | P-Value | Benjamini-Hochberg P-value |
| GOTERM_BP_FAT | GO:0001944~vasculature development | 26 | 2.1E-05 | 2.3E-03 |
| GOTERM_BP_FAT | GO:0048514~blood vessel morphogenesis | 22 | 9.6E-05 | 7.0E-03 |
| GOTERM_BP_FAT | GO:0001568~blood vessel development | 23 | 2.9E-04 | 1.5E-02 |
| GOTERM_BP_FAT | GO:0001525~angiogenesis | 16 | 7.7E-04 | 3.2E-02 |
|  |  |  |  |  |
| Annotation Cluster 11 | Enrichment Score: 3.19 |  |  |  |
| Category | Term | Gene Count | P-Value | Benjamini-Hochberg P-value |
| INTERPRO | IPR013151:Immunoglobulin | 26 | 1.9E-07 | 2.0E-04 |
| UP_SEQ_FEATURE | domain:Ig-like C2-type 1 | 21 | 1.7E-05 | 7.2E-03 |
| UP_SEQ_FEATURE | domain:Ig-like C2-type 2 | 21 | 1.9E-05 | 6.5E-03 |
| INTERPRO | IPR003599:Immunoglobulin subtype | 30 | 2.3E-05 | 4.9E-03 |
| SP_PIR_KEYWORDS | Immunoglobulin domain | 36 | 3.6E-05 | 1.7E-03 |
| INTERPRO | IPR003598:Immunoglobulin subtype 2 | 21 | 1.0E-04 | 1.8E-02 |
| UP_SEQ_FEATURE | domain:Ig-like C2-type 3 | 15 | 1.2E-04 | 3.6E-02 |
| SMART | SM00409:IG | 30 | 1.6E-04 | 8.4E-03 |
| SMART | SM00408:IGc2 | 21 | 4.4E-04 | 1.9E-02 |
| INTERPRO | IPR007110:Immunoglobulin-like | 35 | 7.4E-04 | 7.5E-02 |
| INTERPRO | IPR013783:Immunoglobulin-like fold | 37 | 1.1E-03 | 8.2E-02 |
| UP_SEQ_FEATURE | domain:Ig-like V-type | 12 | 5.1E-03 | 4.6E-01 |
| UP_SEQ_FEATURE | domain:Ig-like C2-type 5 | 7 | 1.4E-02 | 6.5E-01 |
| INTERPRO | IPR013098:Immunoglobulin I-set | 12 | 1.6E-02 | 4.4E-01 |
| UP_SEQ_FEATURE | domain:Ig-like C2-type 4 | 8 | 1.9E-02 | 7.4E-01 |
| UP_SEQ_FEATURE | domain:Ig-like C2-type 6 | 5 | 4.3E-02 | 8.7E-01 |
| INTERPRO | IPR013106:Immunoglobulin V-set | 16 | 5.1E-02 | 6.9E-01 |
| UP_SEQ_FEATURE | domain:Ig-like C2-type 7 | 3 | 1.9E-01 | 9.9E-01 |
|  |  |  |  |  |
| Annotation Cluster 12 | Enrichment Score: 3.11 |  |  |  |
| Category | Term | Gene Count | P-Value | Benjamini-Hochberg P-value |
| GOTERM_BP_FAT | GO:0043069~negative regulation of programmed cell death | 34 | 6.0E-06 | 1.0E-03 |
| GOTERM_BP_FAT | GO:0060548~negative regulation of cell death | 34 | 6.3E-06 | 1.0E-03 |
| GOTERM_BP_FAT | GO:0006916~anti-apoptosis | 24 | 7.0E-06 | 9.6E-04 |
| GOTERM_BP_FAT | GO:0043066~negative regulation of apoptosis | 32 | 2.9E-05 | 2.9E-03 |
| GOTERM_BP_FAT | GO:0010941~regulation of cell death | 56 | 6.7E-05 | 5.6E-03 |
| GOTERM_BP_FAT | GO:0043067~regulation of programmed cell death | 55 | 1.2E-04 | 7.1E-03 |
| GOTERM_BP_FAT | GO:0042981~regulation of apoptosis | 53 | 3.1E-04 | 1.5E-02 |
| GOTERM_BP_FAT | GO:0010942~positive regulation of cell death | 27 | 2.4E-02 | 2.8E-01 |
| GOTERM_BP_FAT | GO:0043065~positive regulation of apoptosis | 26 | 3.5E-02 | 3.4E-01 |
| GOTERM_BP_FAT | GO:0043068~positive regulation of programmed cell death | 26 | 3.8E-02 | 3.5E-01 |
| GOTERM_BP_FAT | GO:0006917~induction of apoptosis | 16 | 2.9E-01 | 8.8E-01 |
| GOTERM_BP_FAT | GO:0012502~induction of programmed cell death | 16 | 2.9E-01 | 8.8E-01 |
|  |  |  |  |  |
| Annotation Cluster 13 | Enrichment Score: 3.09 |  |  |  |
| Category | Term | Gene Count | P-Value | Benjamini-Hochberg P-value |
| GOTERM_BP_FAT | GO:0008219~cell death | 48 | 4.7E-04 | 2.2E-02 |
| GOTERM_BP_FAT | GO:0016265~death | 48 | 5.4E-04 | 2.4E-02 |
| GOTERM_BP_FAT | GO:0012501~programmed cell death | 42 | 6.6E-04 | 2.8E-02 |
| SP_PIR_KEYWORDS | Apoptosis | 27 | 1.3E-03 | 4.6E-02 |
| GOTERM_BP_FAT | GO:0006915~apoptosis | 40 | 1.7E-03 | 5.3E-02 |
|  |  |  |  |  |
| Annotation Cluster 14 | Enrichment Score: 2.96 |  |  |  |
| Category | Term | Gene Count | P-Value | Benjamini-Hochberg P-value |
| GOTERM_MF_FAT | GO:0005125~cytokine activity | 26 | 1.2E-07 | 9.1E-05 |
| INTERPRO | IPR001811:Small chemokine, interleukin-8-like | 10 | 1.8E-05 | 4.7E-03 |
| SP_PIR_KEYWORDS | cytokine | 20 | 2.7E-05 | 1.4E-03 |
| SMART | SM00199:SCY | 10 | 4.3E-05 | 3.1E-03 |
| GOTERM_MF_FAT | GO:0008009~chemokine activity | 10 | 5.4E-05 | 1.3E-02 |
| GOTERM_MF_FAT | GO:0042379~chemokine receptor binding | 10 | 9.0E-05 | 1.6E-02 |
| INTERPRO | IPR002473:Small chemokine, C-X-C/Interleukin 8 | 6 | 1.1E-04 | 1.7E-02 |
| INTERPRO | IPR018048:Small chemokine, C-X-C, conserved site | 6 | 3.2E-04 | 3.7E-02 |
| PIR_SUPERFAMILY | PIRSF500572:small inducible cytokine, A2 type | 4 | 6.5E-04 | 1.0E-01 |
| INTERPRO | IPR001089:Small chemokine, C-X-C | 5 | 1.1E-03 | 9.3E-02 |
| GOTERM_BP_FAT | GO:0007610~behavior | 33 | 1.9E-03 | 5.9E-02 |
| PIR_SUPERFAMILY | PIRSF002522:CXC chemokine | 5 | 2.0E-03 | 2.0E-01 |
| KEGG_PATHWAY | hsa04621:NOD-like receptor signaling pathway | 10 | 2.8E-03 | 1.8E-01 |
| GOTERM_BP_FAT | GO:0042330~taxis | 15 | 4.5E-03 | 1.0E-01 |
| GOTERM_BP_FAT | GO:0006935~chemotaxis | 15 | 4.5E-03 | 1.0E-01 |
| SP_PIR_KEYWORDS | inflammatory response | 9 | 6.1E-03 | 1.1E-01 |
| KEGG_PATHWAY | hsa04060:Cytokine-cytokine receptor interaction | 23 | 7.9E-03 | 3.2E-01 |
| BBID | 109.Chemokine_families | 7 | 8.3E-03 | 3.3E-01 |
| SP_PIR_KEYWORDS | Pyrrolidone carboxylic acid | 8 | 9.7E-03 | 1.4E-01 |
| GOTERM_BP_FAT | GO:0007626~locomotory behavior | 20 | 1.3E-02 | 2.0E-01 |
| SP_PIR_KEYWORDS | chemotaxis | 8 | 1.4E-02 | 1.8E-01 |
| KEGG_PATHWAY | hsa04062:Chemokine signaling pathway | 16 | 3.7E-02 | 6.1E-01 |
| INTERPRO | IPR000827:Small chemokine, C-C group, conserved site | 4 | 6.7E-02 | 7.4E-01 |
| PIR_SUPERFAMILY | PIRSF001950:small inducible chemokine, C/CC types | 4 | 6.7E-02 | 8.8E-01 |
|  |  |  |  |  |
| Annotation Cluster 15 | Enrichment Score: 2.95 |  |  |  |
| Category | Term | Gene Count | P-Value | Benjamini-Hochberg P-value |
| GOTERM_CC_FAT | GO:0043005~neuron projection | 31 | 1.2E-04 | 4.3E-03 |
| GOTERM_CC_FAT | GO:0043025~cell soma | 18 | 7.4E-04 | 1.7E-02 |
| GOTERM_CC_FAT | GO:0030425~dendrite | 16 | 3.7E-03 | 6.5E-02 |
| GOTERM_CC_FAT | GO:0042995~cell projection | 45 | 4.7E-03 | 7.4E-02 |
|  |  |  |  |  |
| Annotation Cluster 16 | Enrichment Score: 2.88 |  |  |  |
| Category | Term | Gene Count | P-Value | Benjamini-Hochberg P-value |
| GOTERM_BP_FAT | GO:0003013~circulatory system process | 24 | 1.2E-06 | 5.0E-04 |
| GOTERM_BP_FAT | GO:0008015~blood circulation | 24 | 1.2E-06 | 5.0E-04 |
| GOTERM_BP_FAT | GO:0003018~vascular process in circulatory system | 11 | 8.1E-05 | 6.4E-03 |
| GOTERM_BP_FAT | GO:0035150~regulation of tube size | 9 | 1.0E-03 | 3.8E-02 |
| GOTERM_BP_FAT | GO:0050880~regulation of blood vessel size | 9 | 1.0E-03 | 3.8E-02 |
| GOTERM_BP_FAT | GO:0042310~vasoconstriction | 4 | 2.8E-02 | 3.0E-01 |
| GOTERM_BP_FAT | GO:0042311~vasodilation | 4 | 8.2E-02 | 5.4E-01 |
| GOTERM_BP_FAT | GO:0006939~smooth muscle contraction | 4 | 1.4E-01 | 6.9E-01 |
| KEGG_PATHWAY | hsa04270:Vascular smooth muscle contraction | 8 | 3.0E-01 | 8.2E-01 |
|  |  |  |  |  |
| Annotation Cluster 17 | Enrichment Score: 2.87 |  |  |  |
| Category | Term | Gene Count | P-Value | Benjamini-Hochberg P-value |
| UP_SEQ_FEATURE | domain:Sema | 9 | 1.0E-05 | 5.2E-03 |
| INTERPRO | IPR001627:Semaphorin/CD100 antigen | 9 | 1.5E-05 | 5.1E-03 |
| SMART | SM00630:Sema | 9 | 3.3E-05 | 3.6E-03 |
| PIR_SUPERFAMILY | PIRSF005526:semaphorin | 6 | 1.7E-04 | 5.5E-02 |
| INTERPRO | IPR003659:Plexin/semaphorin/integrin | 9 | 2.5E-04 | 3.2E-02 |
| SMART | SM00423:PSI | 9 | 5.4E-04 | 1.9E-02 |
| INTERPRO | IPR002165:Plexin | 6 | 5.7E-03 | 2.9E-01 |
| KEGG_PATHWAY | hsa04360:Axon guidance | 14 | 9.4E-03 | 2.4E-01 |
| SP_PIR_KEYWORDS | neurogenesis | 11 | 3.6E-02 | 3.1E-01 |
| INTERPRO | IPR015943:WD40/YVTN repeat-like | 14 | 3.5E-01 | 9.9E-01 |
| UP_SEQ_FEATURE | compositionally biased region:Arg/Lys-rich (basic) | 3 | 4.0E-01 | 1.0E+00 |
|  |  |  |  |  |
| Annotation Cluster 18 | Enrichment Score: 2.70 |  |  |  |
| Category | Term | Gene Count | P-Value | Benjamini-Hochberg P-value |
| GOTERM_BP_FAT | GO:0006875~cellular metal ion homeostasis | 21 | 9.9E-05 | 6.7E-03 |
| GOTERM_BP_FAT | GO:0048878~chemical homeostasis | 39 | 1.4E-04 | 8.5E-03 |
| GOTERM_BP_FAT | GO:0055065~metal ion homeostasis | 21 | 1.8E-04 | 1.0E-02 |
| GOTERM_BP_FAT | GO:0055082~cellular chemical homeostasis | 30 | 5.6E-04 | 2.5E-02 |
| GOTERM_BP_FAT | GO:0050801~ion homeostasis | 31 | 8.5E-04 | 3.4E-02 |
| GOTERM_BP_FAT | GO:0006873~cellular ion homeostasis | 29 | 9.4E-04 | 3.5E-02 |
| GOTERM_BP_FAT | GO:0006874~cellular calcium ion homeostasis | 18 | 9.5E-04 | 3.6E-02 |
| GOTERM_BP_FAT | GO:0030003~cellular cation homeostasis | 22 | 1.2E-03 | 4.2E-02 |
| GOTERM_BP_FAT | GO:0055074~calcium ion homeostasis | 18 | 1.3E-03 | 4.4E-02 |
| GOTERM_BP_FAT | GO:0042592~homeostatic process | 47 | 2.1E-03 | 6.2E-02 |
| GOTERM_BP_FAT | GO:0055080~cation homeostasis | 23 | 2.3E-03 | 6.6E-02 |
| GOTERM_BP_FAT | GO:0030005~cellular di-, tri-valent inorganic cation homeostasis | 19 | 4.0E-03 | 9.6E-02 |
| GOTERM_BP_FAT | GO:0019725~cellular homeostasis | 31 | 6.0E-03 | 1.3E-01 |
| GOTERM_BP_FAT | GO:0055066~di-, tri-valent inorganic cation homeostasis | 19 | 6.8E-03 | 1.4E-01 |
| GOTERM_BP_FAT | GO:0051480~cytosolic calcium ion homeostasis | 8 | 1.9E-01 | 7.7E-01 |
| GOTERM_BP_FAT | GO:0007204~elevation of cytosolic calcium ion concentration | 7 | 2.7E-01 | 8.6E-01 |
|  |  |  |  |  |
| Annotation Cluster 19 | Enrichment Score: 2.63 |  |  |  |
| Category | Term | Gene Count | P-Value | Benjamini-Hochberg P-value |
| GOTERM_MF_FAT | GO:0005539~glycosaminoglycan binding | 16 | 3.2E-04 | 4.6E-02 |
| GOTERM_MF_FAT | GO:0030247~polysaccharide binding | 16 | 8.8E-04 | 1.0E-01 |
| GOTERM_MF_FAT | GO:0001871~pattern binding | 16 | 8.8E-04 | 1.0E-01 |
| GOTERM_MF_FAT | GO:0030246~carbohydrate binding | 27 | 1.3E-03 | 1.2E-01 |
| GOTERM_MF_FAT | GO:0008201~heparin binding | 10 | 1.8E-02 | 5.0E-01 |
| SP_PIR_KEYWORDS | heparin-binding | 7 | 2.8E-02 | 2.6E-01 |
|  |  |  |  |  |
| Annotation Cluster 20 | Enrichment Score: 2.56 |  |  |  |
| Category | Term | Gene Count | P-Value | Benjamini-Hochberg P-value |
| GOTERM_CC_FAT | GO:0000267~cell fraction | 71 | 1.9E-04 | 6.1E-03 |
| GOTERM_CC_FAT | GO:0005626~insoluble fraction | 54 | 2.0E-03 | 3.9E-02 |
| GOTERM_CC_FAT | GO:0005624~membrane fraction | 52 | 2.5E-03 | 4.6E-02 |
| GOTERM_CC_FAT | GO:0005792~microsome | 19 | 1.2E-02 | 1.5E-01 |
| GOTERM_CC_FAT | GO:0042598~vesicular fraction | 19 | 1.5E-02 | 1.7E-01 |
|  |  |  |  |  |
| Annotation Cluster 21 | Enrichment Score: 2.53 |  |  |  |
| Category | Term | Gene Count | P-Value | Benjamini-Hochberg P-value |
| GOTERM_BP_FAT | GO:0032101~regulation of response to external stimulus | 21 | 4.6E-06 | 8.8E-04 |
| GOTERM_BP_FAT | GO:0040017~positive regulation of locomotion | 16 | 6.6E-06 | 9.9E-04 |
| GOTERM_BP_FAT | GO:0050926~regulation of positive chemotaxis | 6 | 7.0E-04 | 2.9E-02 |
| GOTERM_BP_FAT | GO:0050927~positive regulation of positive chemotaxis | 6 | 7.0E-04 | 2.9E-02 |
| GOTERM_BP_FAT | GO:0032103~positive regulation of response to external stimulus | 10 | 8.8E-04 | 3.4E-02 |
| GOTERM_BP_FAT | GO:0050921~positive regulation of chemotaxis | 6 | 5.1E-03 | 1.1E-01 |
| GOTERM_BP_FAT | GO:0048661~positive regulation of smooth muscle cell proliferation | 6 | 6.9E-03 | 1.4E-01 |
| GOTERM_BP_FAT | GO:0050920~regulation of chemotaxis | 6 | 6.9E-03 | 1.4E-01 |
| GOTERM_BP_FAT | GO:0050795~regulation of behavior | 7 | 9.1E-03 | 1.6E-01 |
| GOTERM_BP_FAT | GO:0048520~positive regulation of behavior | 6 | 1.0E-02 | 1.7E-01 |
| GOTERM_BP_FAT | GO:0002687~positive regulation of leukocyte migration | 4 | 2.0E-02 | 2.5E-01 |
| GOTERM_BP_FAT | GO:0001938~positive regulation of endothelial cell proliferation | 4 | 2.0E-02 | 2.5E-01 |
| GOTERM_BP_FAT | GO:0002685~regulation of leukocyte migration | 4 | 4.3E-02 | 3.8E-01 |
| GOTERM_BP_FAT | GO:0048584~positive regulation of response to stimulus | 16 | 4.8E-02 | 4.0E-01 |
| GOTERM_BP_FAT | GO:0050930~induction of positive chemotaxis | 3 | 5.7E-02 | 4.4E-01 |
