# Supplementary material for: Vitamin D Modulates Expression of the Airway Smooth Muscle Transcriptome in Fatal Asthma
Source: PLoS One. 2015 Jul 24;10(7):e0134057. doi: 10.1371/journal.pone.0134057 (PMC4514847; doi:10.1371/journal.pone.0134057)
Supplement: S5 Table — Categories selected were from clusters with enrichment scores >2.50 and with individual Benjamini-Hochberg corrected p-values <0.05 that correspond to known asthma-related structures and processes, plus categories that met these criteria in Table 3. Genes listed were the differentially expressed ones for the corresponding category. (DOCX) [file pone.0134057.s010.docx]

| Annotation Category | Annotation Term | Number of Genes | Genes | Benjamini-Hochberg P-value |
| --- | --- | --- | --- | --- |
| GOTERM_CC_FAT | GO:0031012~extracellular matrix | 43 | *ACAN, ADAMTS10, ADAMTS15, CD248, CHL1, CILP, COL14A1, COL16A1, COL5A3, COL6A6, COMP, CTGF, ENTPD1, F3, FBLN2, FBN1, FGF1, HAPLN1, HMCN1, LAMA1, LAMB3, LAMC2, LOXL1, MAMDC2, MGP, MMP1, NID2, NOV, NTN1, NTNG1, NTNG2, OLFML2A, PCSK6, SLC1A3, SPINT2, TGFB2, TGFB3, THSD4, TNFRSF11B, VEGFA, VIT, WNT10B, WNT11* | 3.7E-06 |
| SP_PIR_KEYWORDS | Immunoglobulin domain | 34 | *ACAN, BOC, CD274, CD83, CHL1, CILP, CNTN1, CNTN3, CRLF1, FSTL5, HAPLN1, HMCN1, IGSF10, IGSF8, KIRREL3, MALT1, NCAM2, NTRK2, OBSCN, PDCD1LG2, PDGFRL, PILRB, PSG1, PSG4, PSG5, PVR, SCN2B, SCN4B, SEMA3B, SEMA3C, SEMA3F, SIRPA, TRIO, VCAM1* | 0.049 |
| GOTERM_BP_FAT | GO:0048545~response to steroid hormone stimulus | 26 | *ALPL, ANGPT1, AURKA, BCL2, CAV2, CCL2, CRYAB, DUSP1, FOS, HMGB2, HMOX1, IDH1, IL6, JUNB, KCNMA1, LDLR, NEFL, PLA2G4A, PRKCA, PTGS2, SOCS3, SST, TGFB2, TGFB3, TNFRSF11B, WFDC1* | 9.7E-04 |
| GOTERM_BP_FAT | GO:0009611~response to wounding | 63 | *ANXA1, AOC3, BCL2, BDKRB1, BDKRB2, C7, CCL11, CCL13, CCL2, CCL7, CCL8, CCNB1, CCR7, CD14, CD40, CD97, CEBPB, CTGF, CXCL1, CXCL2, CXCL3, CXCL6, ENTPD1, F2R, F2RL2, F3, FOS, GPR68, HMCN1, HMOX1, HRH1, IGF1, IGSF10, IL6, IL8, ITGA2, KLF6, MAP2K3, MASP1, MGLL, MST1, MYH10, NEFL, NFKBIZ, NINJ1, NOG, OLR1, P2RY1, PLA2G4B, PLAT, PLAU, PROCR, PXK, S1PR3, SERPINE1, SLC1A3, SYT7, TFPI, TGFB2, TGFB3, THBD, TLR3, TNFRSF1B* | 1.5E-07 |
| GOTERM_BP_FAT | GO:0006979~response to oxidative stress | 21 | *APOE, BCL2, CRYAB, DHCR24, DUSP1, FOS, G6PD, GAB1, GCLM, GJA3, HMOX1, IDH1, OLR1, PLA2G4A, PPP1R15B, PRKCA, PTGS2, SCARA3, SEPP1, SERPINE1, UCP2* | 7.2E-03 |
| GOTERM_BP_FAT | GO:0030324~lung development | 13 | *CAV2, CTGF, DHCR7, FGF1, FGF18, GATA6, HSD11B1, MAPK8IP3, MGP, RBP4, TBX4, TGFB3, VEGFA* | 0.061 |
| GOTERM_MF_FAT | GO:0005125~cytokine activity | 23 | *BMP1, CCL11, CCL13, CCL2, CCL7, CCL8, CLCF1, CX3CL1, CXCL1, CXCL2, CXCL3, CXCL5, CXCL6, GDF5, GDF6, IL33, IL34, IL6, IL8, LIF, TGFB2, TNFRSF11B, VEGFA* | 0.011 |
| GOTERM_MF_FAT | GO:0008009~chemokine activity | 12 | *CCL11, CCL13, CCL2, CCL7, CCL8, CX3CL1, CXCL1, CXCL2, CXCL3, CXCL5, CXCL6, IL8* | 2.1E-03 |
