# Supplementary material for: Vitamin D Modulates Expression of the Airway Smooth Muscle Transcriptome in Fatal Asthma
Source: PLoS One. 2015 Jul 24;10(7):e0134057. doi: 10.1371/journal.pone.0134057 (PMC4514847; doi:10.1371/journal.pone.0134057)
Supplement: S6 Table — Categories selected were from clusters with enrichment scores >2.50 and with individual Benjamini-Hochberg corrected p-values <0.05 that correspond to known asthma-related structures and processes, plus categories that met these criteria in Table 3. Genes listed were the differentially expressed ones for the corresponding category. (DOCX) [file pone.0134057.s011.docx]

| Annotation Category | Annotation Term | Number of Genes | Genes | Benjamini-Hochberg P-value |
| --- | --- | --- | --- | --- |
| GOTERM_CC_FAT | GO:0031012~extracellular matrix | 43 | *ACAN, ADAMTS10, ADAMTS14, ADAMTS15, BMP4, CHI3L1, CILP, COL14A1, COL15A1, COL16A1, COL4A6, COL5A3, CTHRC1, EGFL6, ELN, F3, FGF1, FMOD, GPC1, GPC3, GPC4, HAPLN1, HMCN1, MAMDC2, MFAP4, MFAP5, MMP12, NID2, NOV, NPNT, NTN1, RELN, SCUBE3, SLC1A3, SPARCL1, SPON1, TFPI2, TGFB2, TGFB3, THSD4, TNFRSF11B, VEGFA, WNT2* | 4.6E-03 |
| SP_PIR_KEYWORDS | Immunoglobulin domain | 36 | *ACAN, ALCAM, ALPK2, BOC, CD274, CILP, CRLF1, HAPLN1, HMCN1, JAM2, KIRREL3, KIT, LRIG1, MALT1, MCAM, NEGR1, NFASC, OBSCN, PDCD1LG2, PDGFRL, PSG1, PSG2, PSG4, PSG5, PSG9, PVR, SDK1, SEMA3A, SEMA3B, SEMA3E, SEMA3F, SEMA4B, SIRPA, TEK, UNC5B, VCAM1* | 1.7E-03 |
| GOTERM_BP_FAT | GO:0048545~response to steroid hormone stimulus | 25 | *A2M, ALPL, AQP1, BCHE, BCL2, BMP4, CA9, CCL2, CRYAB, ENO2, FOS, IDH1, IGFBP2, JUNB, KCNMA1, PLA2G4A, PPARG, RARA, RCAN1, SDC1, SOCS3, TGFB2, TGFB3, TNFRSF11B, WNT2* | 2.8E-04 |
| GOTERM_BP_FAT | GO:0009611~response to wounding | 49 | *A2M, ANXA1, AOC3, APOL2, APOL3, BCL2, BDKRB1, C7, CCL11, CCL13, CCL2, CCL7, CD14, CD302, CD36, CD97, CEBPB, CXCL1, CXCL3, CXCL6, DYSF, F10, F2R, F2RL2, F3, FOS, HMCN1, ID3, IGF1, IL8, ITGA2, KLF6, MAP2K3, NOG, NOX4, OLR1, PDPN, PLAU, S1PR3, SDC1, SERPINA3, SERPINB2, SLC1A3, SYT7, TFPI2, TGFB2, TGFB3, TNFAIP6, TNFSF4* | 9.6E-05 |
| GOTERM_BP_FAT | GO:0006979~response to oxidative stress | 13 | *APOE, BCL2, CRYAB, FOS, GAB1, GCLM, IDH1, NQO1, OLR1, PLA2G4A, SCARA3, SDC1, STAT1* | 0.31 |
| GOTERM_BP_FAT | GO:0030324~lung development | 9 | *BMP4, FGF1, HS6ST1, HSD11B1, NFIB, PDPN, TGFB3, VEGFA, WNT2* | 0.37 |
| GOTERM_MF_FAT | GO:0005125~cytokine activity | 26 | *BMP4, CCL11, CCL13, CCL2, CCL7, CXCL1, CXCL12, CXCL3, CXCL5, CXCL6, FAM3C, GDF10, GDF5, GDF6, GREM2, IL32, IL8, INHBB, LIF, SECTM1, TGFB2, TNFRSF11B, TNFSF10, TNFSF15, TNFSF4, VEGFA* | 9.1E-05 |
| GOTERM_MF_FAT | GO:0008009~chemokine activity | 10 | *CCL11, CCL13, CCL2, CCL7, CXCL1, CXCL12, CXCL3, CXCL5, CXCL6, IL8* | 0.013 |
