# Supplementary material for: Vitamin D Modulates Expression of the Airway Smooth Muscle Transcriptome in Fatal Asthma
Source: PLoS One. 2015 Jul 24;10(7):e0134057. doi: 10.1371/journal.pone.0134057 (PMC4514847; doi:10.1371/journal.pone.0134057)
Supplement: S7 Table — (DOCX) [file pone.0134057.s012.docx]

|  |  |  |  |  |  | Fatal Asthma vs. Non-Asthma at Baseline | | Non-Asthma Vitamin D vs. Baseline | | Fatal Asthma Vitamin D vs. Baseline | |
| --- | --- | --- | --- | --- | --- | --- | --- | --- | --- | --- | --- |
| Gene | Locus | Mean FPKM Non-Asthma Baseline | Mean FPKM Fatal Asthma Baseline | Mean FPKM Non-Asthma Vitamin D | Mean FPKM Fatal Asthma Vitamin D | Log2[Fold Change] | Q-value | Log2[Fold Change] | Q-value | Log2[Fold Change] | Q-value |
| *CDC42EP3* | chr2:37870742-37899326 | 12.9 | 21.3 | 29.5 | 44.5 | 0.72 | 6.3E-03 | 1.20 | 1.9E-03 | 1.07 | 1.9E-03 |
| *CEBPB* | chr20:48807119-48809227 | 61.5 | 60.5 | 102.0 | 101.0 | -0.02 | 1.00 | 0.73 | 1.9E-03 | 0.74 | 7.5E-03 |
| *G6PD* | chrX:153759605-153793261 | 90.5 | 145.5 | 167.2 | 145.2 | 0.68 | 3.1E-02 | 0.88 | 1.9E-03 | 0.00 | 1.00 |
| *HSD11B1* | chr1:209859524-209908295 | 7.5 | 8.3 | 17.4 | 17.6 | 0.14 | 1.00 | 1.22 | 1.9E-03 | 1.09 | 1.9E-03 |
| *IL6* | chr7:22766765-22771621 | 27.6 | 25.8 | 14.1 | 20.6 | -0.10 | 1.00 | -0.97 | 1.9E-03 | -0.33 | 0.68 |
| *ITPR1* | chr3:4535031-4889524 | 5.7 | 3.8 | 14.4 | 14.7 | -0.57 | 2.9E-02 | 1.34 | 1.9E-03 | 1.94 | 1.9E-03 |
