# Supplementary material for: Vitamin D Modulates Expression of the Airway Smooth Muscle Transcriptome in Fatal Asthma
Source: PLoS One. 2015 Jul 24;10(7):e0134057. doi: 10.1371/journal.pone.0134057 (PMC4514847; doi:10.1371/journal.pone.0134057)
Supplement: S8 Table — The table contains the number of raw reads for the paired-end samples, the percentage of mapped reads among the total number of raw reads, the percentage of junction spanning reads among the mapped reads, the percentage of mapped bases that mapped to mRNA, the mean insert size of mapped reads, and the slope and R2 values of ERCC spike-in dose response curves. Samples that were not included in differential expression analyses are highlighted in grey. The two samples marked by an asterisk (*) were dropped because of low percentage of junction spanning reads and mRNA bases, and mean insert size. The other three samples highlighted in grey were dropped because their pairs were not successfully sequenced. (DOCX) [file pone.0134057.s013.docx]

| **Sample** | **Raw Reads - R1** | **Raw Reads - R2** | **Raw Reads - Total** | **Mapped Reads (Percent)** | **Junction Spanning Reads (Percent)** | **mRNA Bases (Percent)** | **Mean Insert Size (bp)** | **ERCC Spike-In Dose Response Curve (Slope)** | **ERCC Spike-In Dose Response Curve ( R^2^)** |
| --- | --- | --- | --- | --- | --- | --- | --- | --- | --- |
| S-001167453 | 56539892 | 56539892 | 113079784 | 86.5 | 30.5 | 95.2 | 412.0 | 0.98 | 0.95 |
| S-001167455* | 40111061 | 40111061 | 80222122 | 88.2 | **13.2** | **56.2** | **167.1** | 1.01 | 0.95 |
| S-001167456 | 46633533 | 46633533 | 93267066 | 87.3 | 27.9 | 89.2 | 253.9 | 0.99 | 0.95 |
| S-001167457 | 52465692 | 52465692 | 104931384 | 89.4 | 27.3 | 87.7 | 299.4 | 0.97 | 0.95 |
| S-001167458 | 60836864 | 60836864 | 121673728 | 91.6 | 23.2 | 73.6 | 236.2 | 0.95 | 0.95 |
| S-001167459 | 57289641 | 57289641 | 114579282 | 88.3 | 29.4 | 94.4 | 335.1 | 0.97 | 0.95 |
| S-001167460* | 19945930 | 19945930 | 39891860 | 92.8 | **13.4** | **41.5** | **166.2** | 0.94 | 0.92 |
| S-001167461 | 46381820 | 46381820 | 92763640 | 87.6 | 28.7 | 92.6 | 325.5 | 0.95 | 0.94 |
| S-001167462 | 55089522 | 55089522 | 110179044 | 88.2 | 30.2 | 95.6 | 373.7 | 0.97 | 0.95 |
| S-001167463 | 43348351 | 43348351 | 86696702 | 89.8 | 30.3 | 94.7 | 392.8 | 0.98 | 0.95 |
| S-001167464 | 45572778 | 45572778 | 91145556 | 89.5 | 30.4 | 94.7 | 391.4 | 0.97 | 0.95 |
| S-001167465 | 43608672 | 43608672 | 87217344 | 87.6 | 30.9 | 95.6 | 435.7 | 0.97 | 0.94 |
| S-001167466 | 52710555 | 52710555 | 105421110 | 89.3 | 27.2 | 86.8 | 297.1 | 0.97 | 0.94 |
| S-001167467 | 41590224 | 41590224 | 83180448 | 89.2 | 26.2 | 84.2 | 299.0 | 0.98 | 0.95 |
| S-001167468 | 44855322 | 44855322 | 89710644 | 89.2 | 30.1 | 93.7 | 473.1 | 0.97 | 0.94 |
| S-001167469 | 43673975 | 43673975 | 87347950 | 85.9 | 28.2 | 91.3 | 339.9 | 0.94 | 0.95 |
| S-001167470 | 49773875 | 49773875 | 99547750 | 89.4 | 27.0 | 85.8 | 300.0 | 0.99 | 0.95 |
| S-001167471 | 38714109 | 38714109 | 77428218 | 86.5 | 28.6 | 95.7 | 331.9 | 0.97 | 0.93 |
| S-001167472 | 35197356 | 35197356 | 70394712 | 90.9 | 30.1 | 94.6 | 439.3 | 0.95 | 0.93 |
| S-001167473 | 46333135 | 46333135 | 92666270 | 87.2 | 24.1 | 80.6 | 269.0 | 0.97 | 0.95 |
| S-001167474 | 54350187 | 54350187 | 108700374 | 91.0 | 29.9 | 95.6 | 384.4 | 1 | 0.94 |
| S-001167475 | 42515301 | 42515301 | 85030602 | 90.6 | 31.4 | 96.2 | 524.7 | 0.97 | 0.91 |
| S-001167476 | 40542278 | 40542278 | 81084556 | 89.9 | 29.7 | 94.5 | 425.4 | 0.99 | 0.94 |
| S-001167477 | 39528620 | 39528620 | 79057240 | 89.1 | 29.0 | 95.7 | 345.5 | 0.92 | 0.92 |
| S-001167478 | 51324330 | 51324330 | 102648660 | 92.3 | 27.8 | 87.3 | 326.4 | 0.98 | 0.95 |
| S-001167479 | 42735572 | 42735572 | 85471144 | 89.4 | 29.0 | 95.2 | 382.5 | 1 | 0.95 |
| S-001167480 | 44398255 | 44398255 | 88796510 | 90.6 | 30.7 | 95.4 | 481.9 | 0.98 | 0.94 |
| S-001167481 | 43817779 | 43817779 | 87635558 | 89.4 | 28.8 | 95.4 | 383.5 | 0.96 | 0.95 |
| S-001167482 | 53591705 | 53591705 | 107183410 | 90.7 | 31.1 | 95.2 | 433.1 | 0.98 | 0.95 |
| S-001167483 | 44729685 | 44729685 | 89459370 | 91.2 | 30.9 | 95.6 | 488.4 | 0.99 | 0.94 |
| S-001167484 | 38311567 | 38311567 | 76623134 | 90.7 | 30.3 | 95.5 | 488.2 | 0.95 | 0.94 |
| S-001167485 | 46304528 | 46304528 | 92609056 | 86.8 | 29.4 | 95.6 | 374.8 | 0.96 | 0.95 |
| S-001167486 | 36505524 | 36505524 | 73011048 | 90.2 | 29.4 | 95.8 | 364.5 | 0.95 | 0.94 |
| S-001167487 | 50222734 | 50222734 | 100445468 | 90.9 | 27.6 | 88.6 | 309.6 | 0.97 | 0.95 |
| S-001167488 | 40818165 | 40818165 | 81636330 | 86.1 | 29.9 | 94.8 | 400.1 | 0.98 | 0.95 |
